# Positive electrostatic therapy of metastatic tumors: selective induction of apoptosis in cancer cells by pure charges

**DOI:** 10.1002/cam4.4267

**Published:** 2021-10-09

**Authors:** Ashkan Zandi, Saeid Rafizadeh‐Tafti, Fatemeh Shojaeian, Mohammad Ali Khayamian, Fereshteh Abbasvandi, Mohammad Faranoush, Robab Anbiaee, Sahar Najafikhoshnoo, Parisa Hoseinpour, Sepanta Assadi, Pouyan Katebi, Zahra Davari sh., Shahriar Shalileh, Mohammad Salemizadeh Parizi, Shohreh Vanaei, Mohammadreza Ghaderinia, Hamed Abadijoo, Payam Taheri, Mohammad Reza Esmailinejad, Hassan Sanati, Mohammad Reza Rostami, Reza Sadeghian, Yasin Kordehlachin, S. M. Sadegh Mousavi‐kiasary, Amir Mamdouh, Seyyed Hossein Miraghaie, Hossein Baharvand, Mohammad Abdolahad

**Affiliations:** ^1^ Nanobioelectronic Devices Lab. Cancer Electronics Research Group School of Electrical and Computer Engineering Faculty of Engineering University of Tehran Tehran Iran; ^2^ Nano Electronic Center of Excellence Nanoelectronics and Thin Film Lab. School of Electrical and Computer Engineering Faculty of Engineering University of Tehran Tehran Iran; ^3^ School of Medicine Shahid Beheshti University of Medical Sciences Tehran Iran; ^4^ ATMP Department Breast Cancer Research Center Motamed Cancer Institute ACECR Tehran Iran; ^5^ Pediatric Growth and Development Research Center Institute of Endocrinology and Metabolism Iran University of Medical Sciences Tehran Iran; ^6^ Cardio‐Oncology Research Center Rajaie Cardiovascuar Medical & Research Center Iran University of Medical Sciences Tehran Iran; ^7^ Department of Radiation Oncology Imam Hossein Hospital Shahid Beheshti University of Medical Sciences Tehran Iran; ^8^ SEPAS Pathology Laboratory Tehran Iran; ^9^ Department of Stem Cells and Developmental Biology Cell Science Research Center Royan Institute for Stem Cell Biology and Technology ACECR Tehran Iran; ^10^ Department of Surgery and Radiology Faculty of Veterinary Medicine University of Tehran Tehran Iran; ^11^ Department of Developmental Biology University of Science and Culture Tehran Iran; ^12^ Cancer Institute Imam Khomeini Hospital Tehran University of Medical Sciences Tehran Iran; ^13^ UT&TUMS Cancer electronic Research Center Tehran University of Medical Sciences Tehran Iran

**Keywords:** apoptosis, cancer, chemotherapy, electrostatic charges, noninvasive treatment, nonionizing radiation, radiotherapy, therapy

## Abstract

**Background:**

We discovered that pure positive electrostatic charges (PECs) have an intrinsic suppressive effect on the proliferation and metabolism of invasive cancer cells (cell lines and animal models) without affecting normal tissues.

**Methods:**

We interacted normal and cancer cell lines and animal tumors with PECs by connecting a charged patch to cancer cells and animal tumors. many biochemical, molecular and radiological assays were carried out on PEC treated and control samples.

**Results:**

Correlative interactions between electrostatic charges and cancer cells contain critical unknown factors that influence cancer diagnosis and treatment. Different types of cell analyses prove PEC‐based apoptosis induction in malignant cell lines. Flowcytometry and viability assay depict selective destructive effects of PEC on malignant breast cancer cells. Additionally, strong patterns of pyknotic apoptosis, as well as downregulation of proliferative‐associated proteins (Ki67, CD31, and HIF‐1α), were observed in histopathological and immunohistochemical patterns of treated mouse malignant tumors, respectively. Quantitative real‐time polymerase chain reaction results demonstrate up/down‐regulated apoptotic/proliferative transcriptomes (P21, P27, P53/CD34, integrin α5, vascular endothelial growth factor, and vascular endothelial growth factor receptor) in treated animal tumors. Expression of propidium iodide in confocal microscopy images of treated malignant tissues was another indication of the destructive effects of PECs on such cells. Significant tumor size reduction and prognosis improvement were seen in over 95% of treated mouse models with no adverse effects on normal tissues.

**Conclusion:**

We discovered that pure positive electrostatic charges (PECs) have an intrinsic suppressive effect on the proliferation and metabolism of invasive cancer cells (cell lines and animal models) without affecting normal tissues. The findings were statistically and observationally significant when compared to radio/chemotherapy‐treated mouse models. As a result, this nonionizing radiation may be used as a practical complementary approach with no discernible side effects after passing future human model studies.

## Graphical abstract

The effect of pure electrostatic charges has been investigated on cultured normal and cancerous breast cell lines for the first time. The selective effect of positive electrostatic charges (PECs) on metastatic cell lines and animal tumors has been reported with no effect on normal tissues. PECs induce apoptosis in metastatic breast cancer cell lines and tumors by suppressing proifration pathways. More than 400 mouse models have been tested to investigate the effects of PECs on cancerous tumors and animal organs by different analyses. Survival analyses depict a high survival rate of treated mice with positive electrostatic charges compared to other conventional cancer treatments such as chemotherapy and radiotherapy.

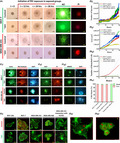

## 1 INTRODUCTION

While radiotherapy (RT), as one of the most desirable and effective local cancer treatments, inhibits tumor growth and progression,^1^, ^2^ post‐radiation side effects, particularly at the treatment site, significantly impact patients’ lifestyles.^3^, ^4^, ^5^, ^6^ Even advanced guided RT methods, such as intensity‐modulated radiotherapy (IMRT)^7^, ^8^ and volumetric‐modulated radiotherapy (VMAT),^9^, ^10^ induce considerable side effects. For example, post‐radiation colitis^11^ and cardiac ischemia^12^ have been reported following IMRT and VMAT in prostate and breast cancers, respectively. Nonionizing radiations, on the other hand, are restricted in their ability to destroy tumors due to their low energy and limited penetration.^13^

The tumor‐treating field, as the most well‐known nonionizing radiative technique, demonstrated a 5‐year survival rate of approximately 30%.^14^ Other than field‐based energy, it appears that new aspects of electron capabilities should be considered for cancer treatment. The selective attraction of malignant cells to external pure positive electrostatic charges (PECs) has been identified and investigated in this study. Examining this attraction at the cellular and clinical levels enables developing a novel complementary tumor therapeutic procedure based on PEC, showing no adverse effect on normal cells. These phenomena might be caused by numerous parameters, including total negative charges of malignant cells,^15^ their abnormal proliferation, and their different signaling pathways.^16^

Additionally, a recent study established the role of intrinsic electrostatic charges in one type of membrane phospholipids in malignant cells, demonstrating that they affect the KRAS and MAPK mitotic pathways.^17^ This study examines the interactions between a floating source of PEC (produced by a rubbing electrostatic generator, Van de Graff generator, 50 kV, accumulating on a sealed metallic patch) and normal and malignant breast cell lines, as well as mouse models with solid metastatic breast tumors. The effects of PEC were investigated by examining the cells’ vital functions, such as adhesion, cytoskeletal assembly, proliferation, and metabolic pathways. PECs were administered to mouse model tumors at various doses and durations.

Moreover, the PEC treatment's efficacy was compared to RT and chemotherapy (ChT) in mouse models (permission was obtained from the ethical committee). Their most vital organs were exposed to PECs, and critical parameters such as serological levels, tumor sizes, quality of life, and possible side effects of PECT (PEC therapy) on their other organs were monitored.

## 2 MATERIALS AND METHODS

### 2.1 Cell culture

The MDA‐MB‐468, MDA‐MB‐231, and MCF‐7 human breast cancer cell lines and the MCF‐10A non‐cancerous breast epithelial cell line were obtained from the National Cell Bank of Pasteur Institute of Iran (NCBI) and Iranian Biological Resource Center (IBRC). MDA‐MB‐468 (NCBI, Cat. No. C208), MDA‐MB‐231 (NCBI, Cat. No. C578), and MCF‐7 (NCBI, Cat. No. Cs135) cells were cultured in Dulbecco's Modified Eagle Medium (DMEM, Sigma, D5648) supplemented with 10% fetal bovine serum (FBS, Gibco, 10270‐106) and 1% penicillin/streptomycin (PenStrep, Gibco, 15140‐172). MCF‐10A cells (IBRC, Cat. No. IBRC C10788) were cultured in DMEM/F12 medium (Sigma, Pro. No. D6421) supplemented with 10% horse serum (Sigma, Pro. No. H1138) and 1% PenStrep (Gibco, Pro. No. 15140‐172). All cell lines were cultured in a standard cell culture incubator (37℃, 5% CO_2_, RH ~95%), with media replenished every 2 days.

### 2.2 Electrostatic stimulation

An electrically isolated metallic patch was placed tangent to the cell culture or on top of the animal's skin/tumor region to stimulate electrostatically. A thin flexible metal sheet is used as an electrostatic patch (aluminum or copper foils). One of the most critical factors to consider is the animals’ range of motion, so the flexible patch was used to keep the animal moving. For preventing current (charge) leakage, the metal sheet was covered entirely with an electrical isolation biocompatible layer called polydimethylsiloxane (PDMS, Dow Corning SYLGARD 184) (Figure S4A). The patch was adhered to the skin with anti‐allergic tape/glue (anti‐allergic surgical tape, Micropore Company) and connected to the electrostatic charge generator via a coaxial cable (Figure S4B) (high‐voltage DC cable, KDK KAWASAKI‐Y 30 kV DC 22AWG). The patches are applied on top of the cell line and mouse model, respectively, as illustrated in Figures S1A and S3A. It is worth noting that various parameters should be monitored during electrostatic stimulation in order to maximize charge accumulation and minimize charge leakage. Initially, the electrostatic patch should be isolated electrically. Regular inspections of the electrostatic patch should be performed to detect instances of charge leakage or current loss. Humidity has a significant effect on the electrostatic patch's discharge. Humidity levels of the animal skin and the surrounding environment should be maintained at low. While keeping the animal skin dry, dry air and atmosphere can cause severe pulmonary problems in animal models. As a result, a trade‐off between leakage ratio and air humidity should exist. Figure S5 depicts tumor destruction rate per applied charge for different applied electrostatic voltages, which proves the effect of charge leakage and decrease in the amount of the applied voltage on tumor destruction rate.

### 2.3 Cell viability assay

The 3‐(4,5‐dimethylthiazol‐2‐yl)‐2,5‐diphenyltetrazolium bromide (MTT) assay was conducted to analyze the anti‐proliferative effects of electrostatic stimulations. The cells were cultured in 12‐well plates. Each well was seeded with 10^5^ cells in a final volume of 500 μl and cultured in a standard cell culturing incubator (37℃, 5% CO_2_, RH ~95%) for 24 h. Electrostatic charges (−1, 0.1, 0.5, and 1 kV) were used to stimulate the cultured cells for 20 h. After the stimulation, 50 μl of the MTT (Sigma‐Aldrich, Pro. No. M2128) solution (5 mg/ml) and 50 μl of DMEM (serum‐free cell media, Sigma, Pro. No. D5648) were added to each well, followed by incubation for 3 hours in the standard cell culturing incubator (37℃, 5% CO_2_, RH ~95%). Afterward, the medium was removed, and 150 μl of dimethyl sulfoxide (DMSO, Sigma‐Aldrich, Pro. No. D4540) was added to each well. The plate was wrapped in foil and shaken gently for 15 min. The absorbance was measured at 570 nm by a Microplate Reader (BioTek ELx808). Negative control (the well containing the medium without any cells) was used for the zero‐absorbance calibration. Data gathered from five independent biological replicates for each test. The results were compared to the positive control cell population and expressed as a percentage of the total cell population.

### 2.4 **Nitrite (** ${\text{NO}}_{2}^{‐}$ **) detection**

The Griess assay (sulfanilamide and NED, Nitrite Assay Kit, Griess Reagent, Sigma‐Aldrich, pro. no. 23479‐1KT‐F) was used to measure nitrite ion (${\text{NO}}_{2}^{‐}$). Nitrite ion is a stable metabolism product of nitric oxide. The Griess reagent was added to the same volumes of the cells’ medium and incubated for 15 min (37℃, 5% CO_2_, RH ~95%). Afterward, the production of azo dyes was investigated using a spectrophotometer (BioTek ELx808) absorbance at 540 nm. Data gathered from five independent biological replicates for each test.

### 2.5 Cell cycle analysis

The treated cells were suspended in trypsin (Gibco, Pro. No. 25200–56) and centrifuged (100*g* for 5 min at room temperature) with the upper cell medium to collect all of the contents of the wells (in order to avoid missing the suspended necrosis cells). The cell pellet was resuspended in phosphate‐buffered saline (PBS, Gibco, Pro. No. 70011‐044), and cold ethanol was added dropwise to the final concentration of 70%. The cell solution was stored on ice for at least 2 h. Afterward, a cocktail of PBS with 100 µg/ml of RNase A, 50 µg/ml of propidium iodide (PI), and optionally 0.1% of Triton X‐100 (PI flow cytometry kit, Abcam, Pro. Co. ab139418) was added. The container was wrapped in foil and stored overnight at 4℃.

Finally, the stored solution was analyzed by a flow cytometer (BD FACSCalibur). The data were analyzed by the FlowJo (v10.5.3) software. The results were gathered from five independent biological replications.

### 2.6 Apoptosis and necrosis detection

The treated cells were suspended in trypsin (Gibco, Pro. No. 25200‐56) and centrifuged (100*g* for 5 min at room temperature) with the upper cell medium to collect all of the contents of the wells (in order to avoid missing the suspended necrosis cells). The cell pellet was resuspended in phosphate‐buffered saline (PBS, Gibco, Pro. No. 70011‐044), and a mixture containing 100 µl of cells, 100 µl of incubation buffer with 2 µl of Annexin‐V (1 mg/ml, Annexin V‐FITC Apoptosis Staining, Abcam, Pro. Co. ab14085), and 2 µl of propidium iodide (1 mg/ml, PI, Abcam, Pro. Co. ab139418) was prepared. The mixture was wrapped in a foil and incubated on an orbital shaker (30 rpm, Vibromix 60, Domel). Afterward, the mixture was centrifuged (100*g* for 5 min at room temperature), and the upper medium was removed to avoid background color and unbound Annexin V and PI. The cell pellet was resuspended in phosphate‐buffered saline and analyzed by a flow cytometer (BD FACSCalibur). The data were analyzed by the FlowJo (v10.5.3) software. The gating strategy is demonstrated in Figure S6. Data gathered from five independent biological replications for each test.

### 2.7 Spheroid formation procedure

MDA‐MB‐231 and MCF‐7 cell lines were trypsinized and suspended using trypsin (Gibco, pro. no. 25200‐56), and they were then centrifuged (100*g* for 5 min at room temperature). The suspended cells were counted using a hemocytometer lam (Marienfeld), and several droplets of 20 µl suspended cells (~1 × 10^3^ cells) were deposited on the lid of a cell culture dish. The lid was returned to the cell culture dish (SPL Life Sciences), and the droplet hanged upside down and incubated for 2 days in a standard cell culturing incubator (37℃, 5% CO_2_, RH ~95%). Following that, the desired spheroids were collected and added to the collagen matrix (~100–150 µm, using an inverted microscope; grown spheroids should have a tight and compact spherical shape; disintegrated spheroids and those with loose aggregations are not suitable) (type I rat tail, Corning). The spheroid and collagen mixtures were incubated for 30 min (37℃, 5% CO_2_, RH ~95%). After gel cross‐linking, the cell culture medium was added to the combination.^18^, ^19^

### 2.8 Immunofluorescent microscopy of cell lines

The immunofluorescent microscopy of cytoskeletal actin microfilaments was provided using an inverted confocal microscopy system (Leica, TCS SP5). Treated (stimulated) and non‐treated cells fixed by 4% methanol‐free formaldehyde (Sigma‐Aldrich, Pro. No. 489417, Merck, Pro. No. 1‐27‐3228) solution and gently washed three times with phosphate‐buffered saline (PBS, Gibco, Pro. No. 70011‐044). The fixed cells were permeabilized using 0.2% Triton‐X100 (Sigma‐Aldrich, Pro. No. T8787) in PBS for 20 min and then gently washed three times with PBS. Blocking was provided using 1% bovine serum albumin (BSA, Sigma‐Aldrich, Pro. No. A5611) diluted with PBS in the incubator (37℃, 5% CO_2_, RH ~95%) for 1 h. In the next step, BSA was aspirated, and actin dye Alexa Fluor^®^ 488 Phalloidin (Invitrogen, Cat. No. A12379) was added to the cells and maintained for 45 min in an incubator (37℃, 5% CO_2_, RH ~95%). Propidium iodide dye (PI, Invitrogen, Cat. No. P3566) was used to stain the cell nucleus. In the final step, inverted confocal microscopy (Zeiss, LSM 800) at 1000× magnification was used to obtain the cell images.

### 2.9 RNA isolation and RT‐qPCR analysis

According to the manufacturer's instructions, total RNA was extracted from frozen tissue using the TRIzol reagent (Tiangen, Cat. No. 15596‐026). The RNAs were extracted by CCl_3_ (Aladdin, SEKR‐0025) and dissolved in DEPC water (Sigma‐Aldrich, Pro. No. 693520). The RNA concentration was measured using a UV spectrophotometer (NanoDrop One Microvolume UV‐Vis spectrophotometer, Thermo Fisher Scientific). cDNA was synthesized using reverse‐transcribed RNA by a kit (Tiangen, Cat. No. 499278), following the manufacturer's procedure. The amount of RNA used was 1000 ng in 20 µl. The reverse transcription reaction was performed at 37℃ for 15 min, and the inactivation of the reverse transcriptase condition was at 85℃ for 15 s. Real‐time polymerase chain reaction (PCR) was performed using a Thermal Cycler Dice Real‐Time PCR System (TaKaRa, TP950). Activating the DNA polymerase was performed at 95℃ for 5 min, followed by 40 cycles of a two‐step PCR (95℃ for 10 s and 60℃ for 30 s), and a final extension at 75℃ for 10 min, and storage at 4℃. The primers used for SYBR Green real‐time RT‐PCR are reported in Table S1 (Thermo Fisher Scientific). Both oligo and random hexamer primers were used to convert most of the RNA to cDNA. A dissociation curve analysis of P21, P27, P53, vascular endothelial growth factor (VEGF), vascular endothelial growth factor receptor (VEGF‐A), integrin α5, and GAPDH showed a single peak. The mean Ct of the gene of interest was calculated from triplicate measurements and normalized with the mean Ct of a control gene, that is, GAPDH.^20^ The obtained Ct values for the controls were between 19 (for the lowest) and 30 (for the greatest), and the samples were normalized to them.

### 2.10 Tumor formation in animal models

Female inbred BALB/c mouse models were purchased at the age of 4–5 weeks from the Pasteur Institute of Iran. They were kept at a temperature of 22–27℃ with a 12‐h light/dark cycle in a pathogen‐free isolation facility that was meticulously designed. They were allowed to adapt to the environment for 1 week before experimentation. The Animal Ethics Committee approved the entire procedure. A total of 1 × 10^6^ 4T1 cells/100 μl in the logarithmic growth phase were subcutaneously (sub‐Q) injected into the flank region of the BALB/c mouse models. Once the tumor mass was established on day 12 (tumor volume ~800 mm^3^), the mouse models were randomly assigned to various groups (10 mouse models per group). The mouse models were exposed to 1–5 kV (16–90 nC) electrostatic stimulation for 12 days. Tumor size was determined twice weekly using a portable sonogram until the mouse models were euthanized according to the standard reported guidelines.^21^ Different endpoints were used to determine when the mouse models should be euthanized, discussed in Tables S2 and S3 in detail. The majority of PECT‐treated mouse models were maintained until their final days of life (approximately 1 year) to determine the long‐term effects of PECT, the likelihood of recurrence, and the survival rate of treated mouse models (Tables S2 and S3). The RT, ChT, NECT, and control groups were all maintained until natural death (Tables S2 and S3). The volume of the tumor was determined using the following equation^22^, ^23^:

$$\text{Tumor volume}=\left(\frac{4}{3}\right)\times \pi \times \left(\frac{\text{Length}}{2}\right)\times \left(\frac{\text{Length}}{2}\right)\times \left(\frac{\text{Depth}}{2}\right).$$

### 2.11 Histological staining

The desired tissues were fixed in 10% neutral buffered formalin (NPF, fixative, Sigma‐Aldrich, HT501128) for 10 min. Afterward, the fixed tissues were immersed consecutively in the following solutions: 1%–10% NPF for 2 h, 2%–10% NPF for 2 h, 3%–60% ethanol for 1 h, 4%–70% ethanol for 1 h, 5%–80% ethanol for 1 h, 6%–90% ethanol for 2 h, 7%–96% ethanol for 2 h, 8%–99% ethanol (Sigma‐Aldrich, Pro. No. 1117270500) for 2 h, 9‐xylene (Sigma‐Aldrich, Pro. No. 534056) for 1 h, 10‐xylene (Sigma‐ Aldrich, Pro. No. 534056) for 2 h, 11–60℃ paraffin (Paraplast Plus, Sigma‐Aldrich, Pro. No. P368) for 2 h, and 12–60℃ paraffin (Paraplast Plus, Sigma‐Aldrich, Pro. No. P3683) for 2 h. Following that, the fixed tissues were embedded in paraffin (the final step in the tissue processing procedure) and serially sectioned into 2‐µm‐thick sections using a manual rotary microtome (Leica). In the next step, the sections were immersed in Mayer's Hematoxylin (Abcam, Pro. Co. ab220365) for 10 min, followed by rinsing with cool distilled water for 5 min in a Coplin jar. Eosin staining was performed by dipping the sample in 0.5% eosin dye (Abcam, Pro. Co. ab246824) 12 times, followed by immersion in distilled water. Finally, the samples were dipped in 50% and 70% ethanol, 10 times each, and equilibrated in 95% and 100% ethanol for 30 s and 1 min, respectively. The samples were dipped in xylene as long as needed. For conducting additional qualitative pathological studies, each slide was photographed at magnifications of 200× and 400× using a camera coupled with the microscope (Olympus‐BX51).

### 2.12 Immunohistochemical staining

The paraffin‐embedded tissues were cut into 2‐µm‐thick sections using a manual rotary microtome (Leica). Afterward, the sections were rehydrated in Coplin jars as follows: 1‐xylene (Sigma‐Aldrich, Pro. No. 534056) for 3 min, 2‐xylene for 3 min, 3‐1:1 xylene:100% ethanol (Sigma‐Aldrich, Pro. No. 1117270500) for 3 min, 4%–100% ethanol for 3 min, 4%–100% ethanol for 3 min, 5%–95% ethanol for 3 min, 6%–70% ethanol for 3 min, and 7%–50% ethanol for 3 min. After rehydrating the slides, they were placed under cold tap water to remove any remaining ethanol. Additionally, sections should never be allowed to dry to avoid non‐specific antibody binding and thus increased background staining. In the next step, the endogenous peroxidase blocking was provided by 3% hydrogen peroxidase to avoid high non‐specific background staining. The sections were treated with mouse anti‐human monoclonal Ki67, P53, HIF‐1α, and CD‐31 (all from Abcam, Pro. Co. ab279657, ab176243, ab8366, ab9498, respectively) antibodies (1:100), diluted in phosphate‐buffered saline containing 0.1% TWEEN‐20 (PBST, Sigma‐Aldrich, pro. no. 524653) and 5% bovine serum albumin (BSA, Sigma‐Aldrich, Pro. No. A3294). The sections were incubated overnight at 4℃, followed by treating with secondary antibodies (1:100), avidin‐biotin‐peroxidase (Abcam, Pro. Co. ab64212), diaminobenzidine (DAB, ScyTek Laboratories, ACK500), and mouse secondary antibody (Jackson Immuno Research Labs). In the final step, hematoxylin (Richard‐Allen Scientific, Cat. No. 7211) was used as the counterstain.

For performing confocal imaging from of the animal tissues, prior to doing the staining such as described above, the prepared slices of the tissues were held at 70℃, followed by immersion in xylene (Sigma‐Aldrich, pro. no. 534056) for 5 min to remove the paraffin from the frozen tissues.

### 2.13 PET scan procedure

Positron emission tomography (PET) is a nuclear imaging technique that uses a radioactive dye to trace the movement of a radioactive dye within living organs. PET scanning enables the monitoring of a cell's metabolic activities. Before performing a PET scan on the mouse models, they were fasted overnight (for at least 6 h). Fluorodeoxyglucose (^18^F‐FDG) was used as the radioactive dye, that is, the radiotracer. ^18^F‐FDG was provided by the Department of Radiation Oncology at the Cancer Institute of Tehran University of Medical Sciences (TUMS). About 300 µCi of ^18^F‐FDG was administrated to the mouse models by intravenous injection of the tail vein. Following that, the mouse models were given 40 min to determine the radiotracer's non‐specific bounds (to avoid false‐positive results). Consequently, 30 µl of a xylazine/ketamine mixture (10 mg/kg xylazine and 90 mg/kg ketamine) was intraperitoneally injected into the mouse models, and they were fixed in the prone position for imaging. Using a PET system (Xtrim PET, PNP Co), the static single‐frame scan was conducted for 10 min. A ramp filter was used to rebuild the images. The resolution used was approximately 2.1 mm, and pixel sizes were determined as 2 × 2 × 1.0 mm^3^.

### 2.14 RT and ChT regimens

Three groups of 10 mouse models with 4T1 tumors were used (10 biological replicates). They were randomly assigned to different treatment groups on day 12 of tumor induction. Paclitaxel (PTX) (Altaxel, Alkem Laboratories Ltd.) was administered through an intraperitoneal bolus injection at a total dose of 20 mg/kg/week to the ChT group (every 7 days for three continuous weeks). Daily abdominal swelling, abdominal distension, regular activity, weight loss, muscle wasting, and any other signs or symptoms of toxicity were assessed.^23^, ^24^, ^25^ The other two groups received RT in two fractions of 1 and 2 Gy each at 48‐h intervals. The animals were then anesthetized intravenously with xylazine/ketamine (10 mg/kg xylazine and 90 mg/kg ketamine). A 6‐MV linear accelerator gave the tumor irradiation at a 200 cGy/min rate using gamma rays from a Co60 source (Theratron 780C, Theratronix); the treatment was repeated after 48 h.^26^, ^27^, ^28^, ^29^ All mouse models were followed until recurrence of tumors or death.

### 2.15 Data analysis and statistics

All the data are expressed as mean ± SD. Each dataset's normal distribution was determined using one‐sample Kolmogorov–Smirnov test and the datasets that lacked normal distribution were normalized in SPSS using two‐step approach.^30^ The level of significance was set to *p* < 0.05. Kaplan–Meier curves were analyzed with the log‐rank test. The differences between the experimental groups were evaluated using one‐way ANOVA followed by Tukey's honest significance differences as a post hoc multiple comparison test and independent *t*‐tests were used when only two groups were compared. SPSS version 25.0 and GraphPad Prism version 8.4.0 were used to conduct the statistical analyses. The graphs were plotted using GraphPad Prism version 8.4.0 and OriginPro version 2018 software.

## 3 RESULTS

### 3.1 In vitro effects of PECs on normal and different grades of cancerous breast cell lines in 2D and 3D spheroid shapes

**FIGURE 1:**
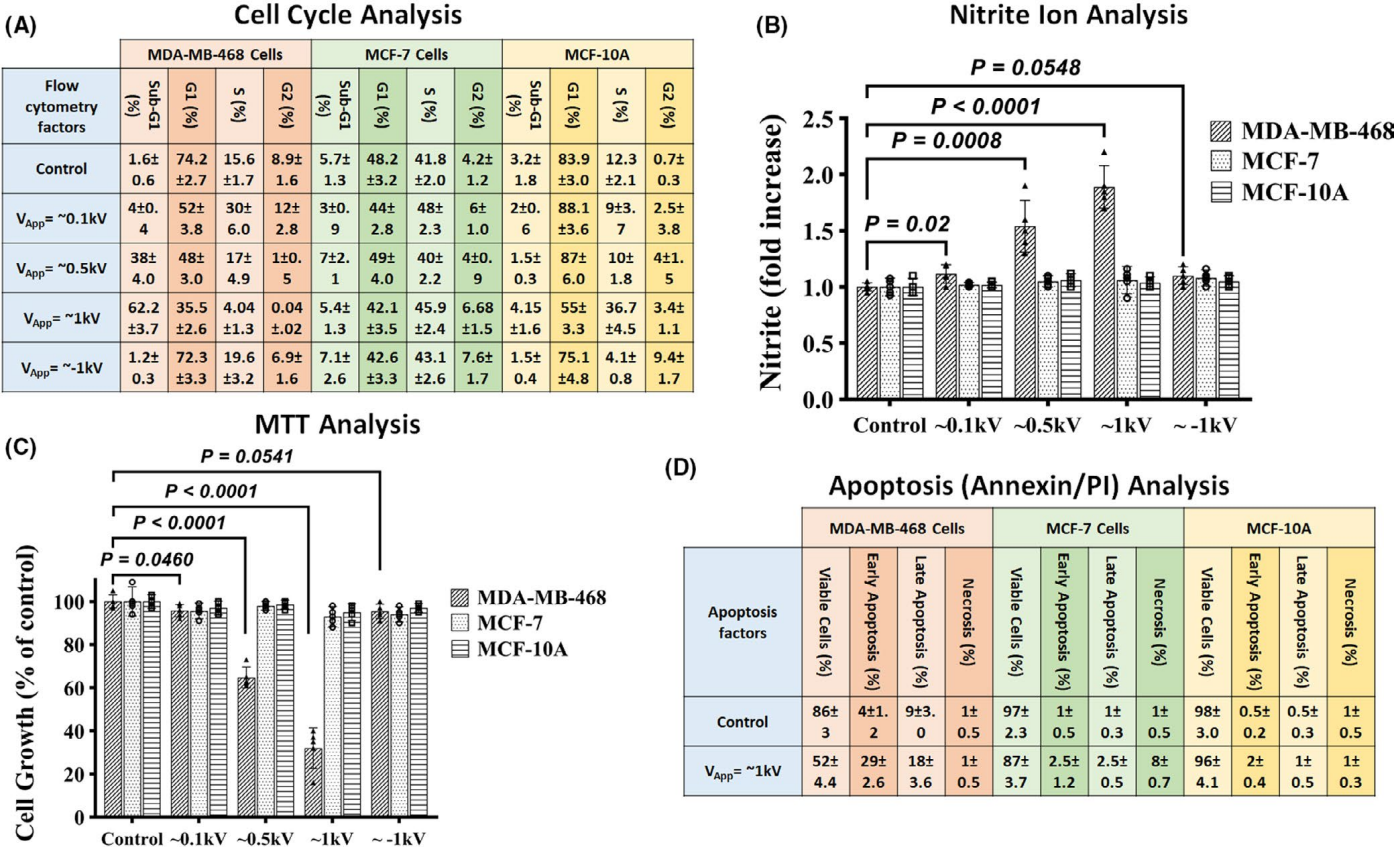
(A) Cell cycle analysis of samples with varying levels of stimulation in the MCF‐7, MCF‐10A, and MDA‐MB‐468 cell lines. The table summarizes the cell cycle analysis performed using flow cytometry. As can be seen, metastatic cells exhibit significantly higher apoptotic rates than normal cells. In MDA‐MB‐468, the difference between the control and exposed groups was statistically significant (*p* < 0.0001, independent *t*‐test). The difference between the groups in both MCF‐7 and MCF‐10A was not significant (*p* > 0.05, independent *t*‐test). All the data are shown as mean ± SD. Data gathered from five independent biological replicates of each test. (B) Nitrite ion (${\text{NO}}_{2}^{‐}$) analysis results. Higher levels of ${\text{NO}}_{2}^{‐}$ in the cellular media could be a signal of rising apoptotic levels. As shown, greater ${\text{NO}}_{2}^{‐}$ levels are mostly seen in metastatic cells (independent *t*‐test). Data gathered from five independent biological replicates of each test. (C) MTT analysis results. MTT showed a significant decrease in the viability of stimulated metastatic cells as a widespread viability assessment method (independent *t*‐test), while for the other two groups of cells, no destructive effects were observed. Data gathered from five independent biological replicates of each test. (D) On control‐ and PEC‐stimulated samples, Annexin/PI analysis was performed. The percentages of cells undergoing early and late apoptosis are depicted. It is possible to observe a selective increase in apoptotic levels in metastatic cells, with a greater emphasis on early apoptotic increment. In MDA‐MB‐468, the difference between the control and exposed groups was significant (*p* < 0.0001, independent *t*‐test), except for necrosis. The difference between groups in both MCF‐7 and MCF‐10A was not significant (*p* > 0.05, independent *t*‐test). All the data are shown as mean ± SD. Data gathered from five independent biological replicates of each test

Initially, the effects of positive and negative electrostatic charges (PECs and NECs) generated by rubbing electrostatic charge generator (~1.6 nanocoulomb, nC, in steady‐state) on four different types of human breast cell lines ranging from normal to highly malignant were investigated (method; Figure S1A). In this case, the charged patch was placed on top of the microwells containing the cells for 24 h. The vitality of the cells was then determined using the flow cytometry assay (Figure 1A & Figure S1C). The results indicated that neither PECs nor NECs (created by rubbing electrostatic charge generator, RECG, at varying intensities) had a detrimental effect on the non‐cancerous breast cell line (MCF‐10A) (Figure 1A, yellow column). The ratio of apoptotic cells (sub‐G1 phase) in triple‐positive (ER+, PR+, and HER‐2/neu+) cancerous breast cell lines (MCF‐7) exposed to PECs was rarely increased, and no changes were observed in MCF‐7 cells exposed to NECs (Figure 1A, green column). In comparison, the apoptotic ratio of a metastatic triple‐negative (ER−, PR−, and HER‐2/neu−) breast cancer cell line (MDA‐MB‐468) exposed to PECs (at a 0.5 kV intensity) was approximately 38%. By increasing the concentration of PECs to 16 nC (intensity of 1 kV), the ratio of apoptotic MDA‐MB‐468 cells was increased to 62% (Figure 1A, light red column). NECs, on the other hand, did not affect MDA‐MB‐468 cells (Figure 1A, light red column).

Additionally, the effects of electrostatic charges on ${\text{NO}}_{2}^{‐}$ production (as an indicator of the apoptotic activation pathway) in normal and cancer cells were investigated. PECs with a power of ≥ ~0.5 kV (≥ ~8 nC) increased ${\text{NO}}_{2}^{‐}$ production significantly only in MDA‐MB‐468 cells, whereas NECs had no additive effect on ${\text{NO}}_{2}^{‐}$ production in any of the assayed cells (Figure 1B).

The MTT assay was used to determine the effect of PECs on cell growth (from 1.6 to 16 nC). PECs with intensities greater than ~0.5 kV were found to significantly inhibit cell growth in MDA‐MB‐468 cells (Figure 1C).

The Annexin/PI assay was used to quantify the actual apoptosis rate in the cell lines^31^, ^32^, ^33^ after 24 h of exposure to PECs (~1 kV) (Figure 1D). PECs induced negligible apoptosis in MCF‐10A (~3% increase in apoptosis: 2% early and 1% late apoptosis) and MCF‐7 cells (~5% increase in apoptosis: 2.5% early and late apoptosis, respectively), but highly significant apoptosis in metastatic MDA‐MB‐468 cells after 24 h (~47% increase in apoptosis: 29% early and 18% late apoptosis) (Figure 1D).

**FIGURE 2:**
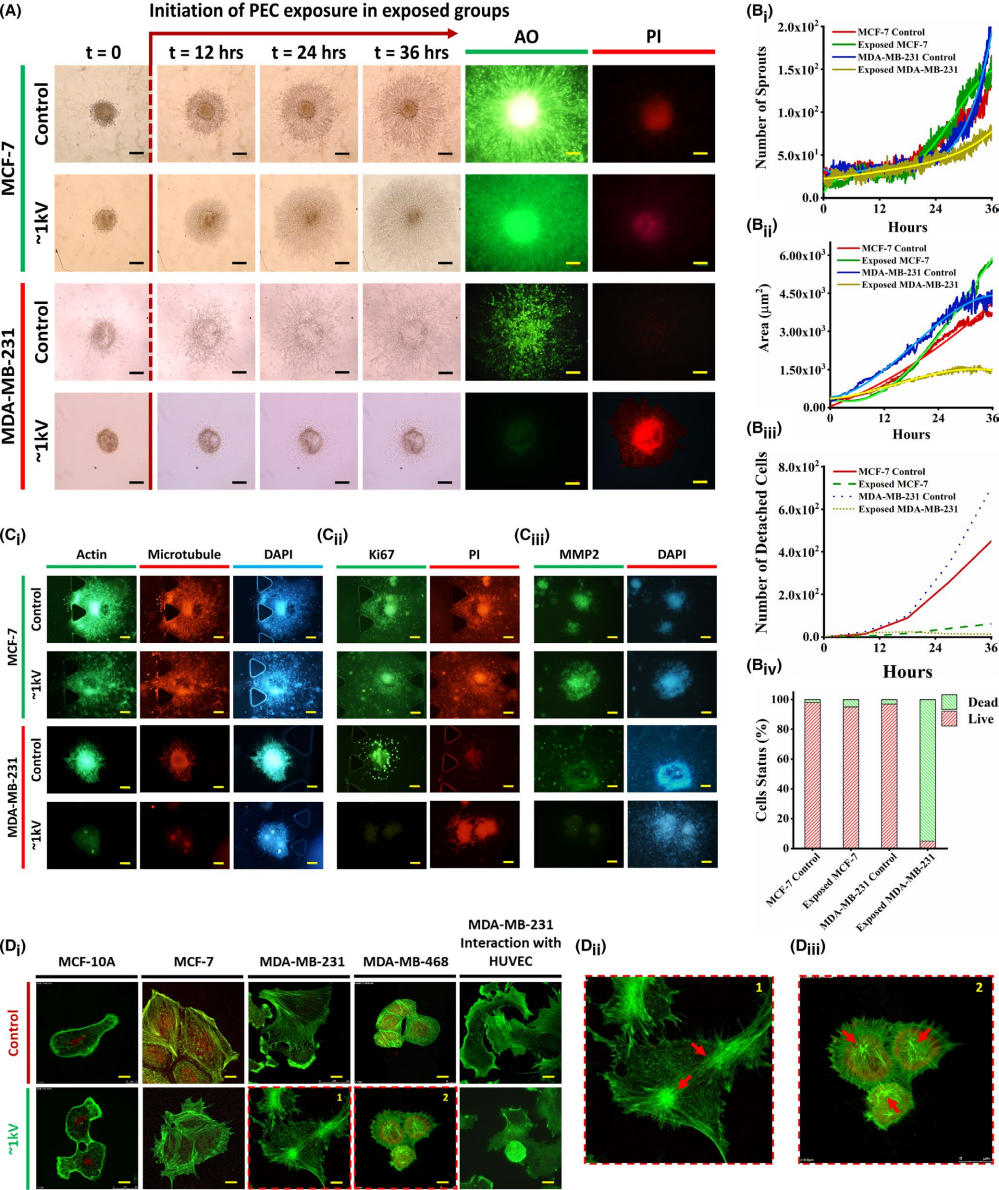
(A) Time‐lapse imaging of 3D spheroids of the MCF‐7 and MDA‐MB‐231 cell lines. PEC exposure started 12 h after primary spheroid implantation in 3D spheroid formation culture. Time‐lapse imaging provides images of spheroids at 12‐h intervals. AO and PI (live and dead) show drastic effects of PEC exposure on the metastatic MDA‐MB‐231 cell line. However, there is no observable effect on the MCF‐7 cell line. Scale bars are set to 100 µm. Time‐lapse imaging was repeated 10 times. Moreover, hrs stand for hours. (B_i_, B_ii_, B_iii_, B_iv_) Comparative analysis of the spheroid area, the number of sprouts, the number of detached cells, and the cell status of the exposed and control spheroids using image processing. As indicated, the exposed MDA‐MB‐231 cells were affected most significantly by PEC. (C_i_, C_ii_, C_iii_) Fluorescent imaging of the exposed and control spheroids of both MCF‐7 and MDA‐MB‐231 cell lines considering cytoskeletal, proliferation, and invasion markers, respectively. The scale bars are set to 100 µm. (D_i_, D_ii_, D_iii_) Confocal microscopy images of the cell lines in the control group and the group stimulated by PEC at ~1 kV for 24 h. A significant change in the direction of tension in actin filaments is observable in exposed metastatic cells compared to the control. The scale bars are set to 10 µm. The pointed arrows indicate the contracted actins in both MDA‐MB‐468 and MDA‐MB‐231 cell lines. AO, acridine orange; PEC, positive electrostatic charge; PI, propidium iodide

The following step involved forming breast triple‐positive and triple‐negative cancer cells in the architecture of a 3D spheroid (method: spheroid formation) to study the effects of PEC in a more realistic model of a tumor. Three invasion‐associated factors of spheroids were analyzed: area (AR), number of formed sprouts (NS), and number of detached/migrated cells from the spheroid (NDC) (Figure S1D_i_). In comparison to the control spheroids, time‐lapse imaging revealed only minor changes in the growth of the MCF‐7 spheroid exposed to PECs (16 nC, 1 kV) (Figure 2A), whereas the MDA‐MB‐231 spheroid experienced a drastic reduction in expansion after <24 h of PECs exposure (Figure 2A, Video S1). Additionally, acridine orange (AO), a fluorescent dye commonly used to monitor cellular vitality,^34^ demonstrated a significant decrease in the number of live cells in the exposed MDA‐MB‐231 spheroids (Figure 2A). Furthermore, live‐dead (AO)/dead (PI) fluorescent imaging revealed a significant decrease in the vitality of post‐exposed MDA‐MB‐231 cells, whereas the MCF‐7 spheroid retained its vitality following exposure (Figure 2A). In comparison to the control counterparts, Figure 2B_i_ demonstrated that 24 h of exposure to PECs did not affect the AR of the MCF‐7 spheroid, whereas the AR of the MDA‐MB‐231 spheroid was suppressed entirely. AR is a good indicator of the tumor's growth rate. This suppression of MDA‐MB‐231 cell proliferation following PEC treatment is highly correlated with the activated apoptotic factors in these cells. Additionally, the NS of exposed MDA‐MB‐231 spheroid was significantly reduced, whereas it remained unchanged in exposed MCF‐7 spheroid (Figure 2B_ii_, Video S2). NS is a good indicator of the tumor's mitotic activity, and this decrease in the treated MDA‐MB‐231 spheroids demonstrates PEC's ability to suppress uncontrolled cancerous functions in triple‐negative malignant breast cells. As a known malignancy parameter of a spheroid,^23^, ^24^ NDC is in direct correlation with migrative and invasive properties of tumor cells. It was reduced in both MCF‐7 and MDA‐MB‐231 spheroids that have been exposed to PECs (Figure 2B_iii_). However, the level of NDC reduction in the treated MDA‐MB‐231 spheroid was very significant.

Actins‐ and microtubules‐conjugated fluorescent proteins were expressed in viable cells (revealing the architecture of the spheroid^23^, ^24^), but not in the exposed MDA‐MB‐231 spheroid (Figure 2C_i_, DAPI was stained as cell indicator). In exposed MDA‐MB‐231 spheroids, the expression of the Ki67 immunofluorescent marker (as an independent proliferation factor) was significantly decreased (Figure 2C_ii_). Mitochondrial membrane potential 2, a well‐characterized protein expressed in cancer cells’ invadopodia and critical for invasion,^37^ was significantly downregulated in the exposed MDA‐MB‐231 spheroid, whereas its expression was unaffected in the exposed MCF‐7 spheroid (Figure 2C_iii_).

Finally, comparative confocal microscopy images of all phenotypes of single breast cells taken before and after exposure to PECs (24 h, ~1 kV) revealed no discernible changes in actin assembly of MCF‐10A and MCF‐7 cells (Figure 2D). Meanwhile, triple negative malignant cells, MDA‐MB‐231 and MDA‐MB‐468, exhibited remodeled actins with an abnormal tendency for these actins to be contracted (Figure 2D). Such disparate responses to PECs by triple‐positive and triple‐negative breast cancer cells warrant additional study. One possible explanation for the MDA‐MB‐231 cells’ strong apoptotic response to PECs compared to MCF‐7 cells is the expression of the Erbb2 protein. This charged and polarized protein enhances the cells’ response to an external electric field. MDA‐MB‐231 cells have been shown to express a significant amount of this protein and to respond normally to an electric field, whereas MCF‐7 cells do not express this protein.^38^

To evaluate invasive functions of malignant cells after exposure to PECs, invasion assay on human umbilical vein endothelial cells (HUVECs) by control and treated (PECs: 8 nC, 0.5 kV, for 6 h) MDA‐MB‐231 cell lines were investigated for 24 h. The MDA‐MB‐231 cells were chosen for this assay as they show more invasive functions than MDA‐MB‐468 cells because they have a higher flavin adenine dinucleotide to nicotinamide adenine dinucleotide ratio.^39^, ^40^ Both control and treated cells were detached from the cell culture plates and then interacted with pre‐cultured HUVECs individually. Comparative confocal images from this assay (for 24 h) demonstrated that MDA‐MB‐231 cells, which had been exposed to PECs, completely lost their invasion ability (Figure 2D). They did not spread through HUVECs and exhibited no invadopodia inside the endothelial cells.^40^

### 3.2 In vivo inhibition of mammary tumor growth by PECs in mouse models

**FIGURE 3:**
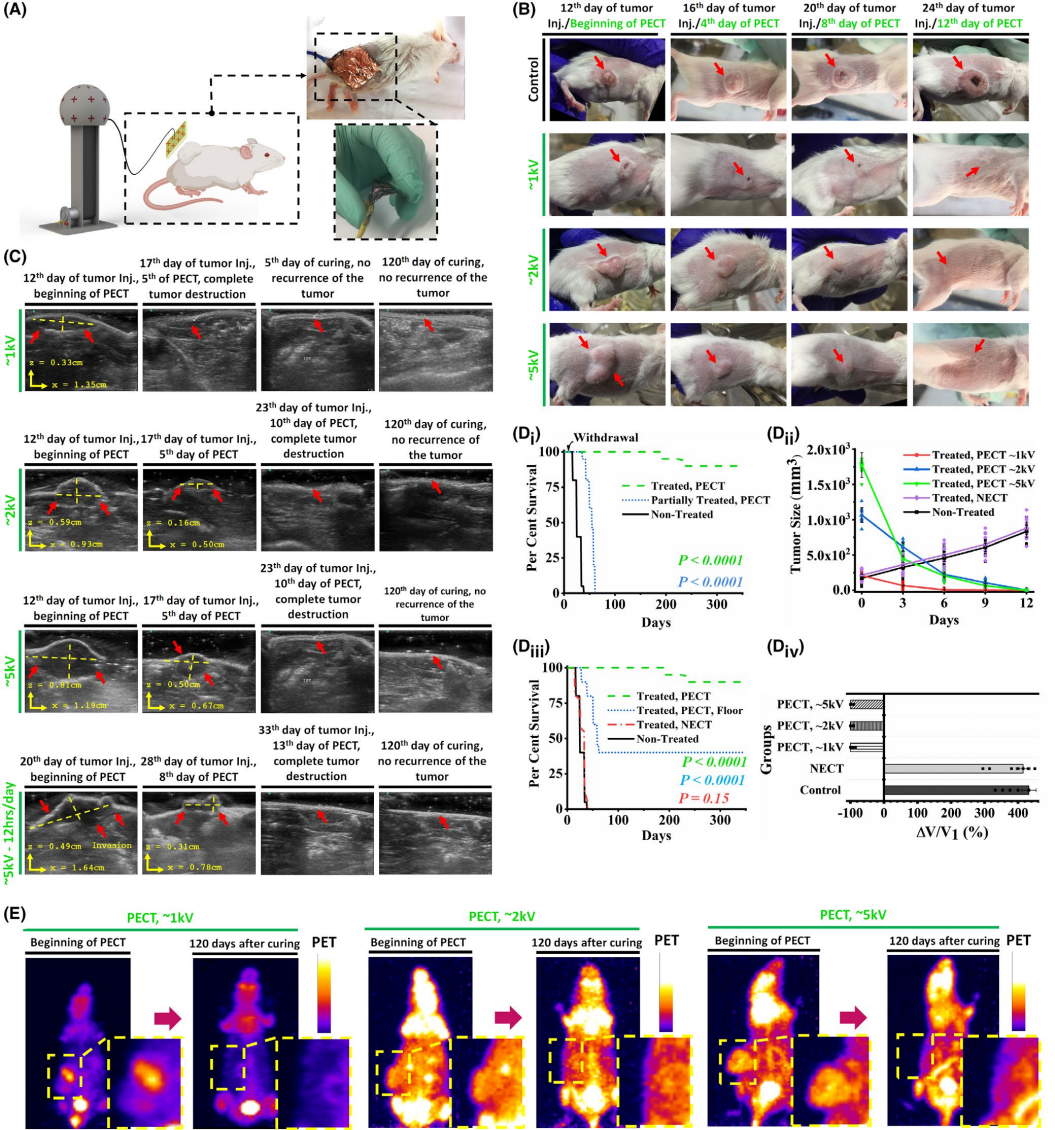
(A) Schematic of the exposure system on the mouse models’ skin with the optical image of the flexible electrical isolated patch attached to the skin of the mouse models using medical‐grade glue. (B) Optical images of the control and exposed mouse models. Significant reduction and destruction of the tumors of the exposed mouse models are demonstrated. The mouse models with larger tumor sizes were exposed to higher levels of PECs. (C) Sonographic images of the tumors in exposed mouse models at the beginning, middle, and end of the experiment. When exposed mouse models are compared to control models, significant tumor growth inhibition in terms of size is observed. For more intense exposures, higher levels of inhibition are observed. (D_i_) Kaplan–Meier survival curves of different mouse models groups. PEC was withdrawn for the partially exposed group after 10 days (log‐rank test). These tests were performed in more than 400 mouse model which allocated in different groups randomly. (D_ii_) Tumor size alterations in the five groups of the study. Higher levels of PECs show higher rates of tumor degradation. The cross‐section of the tumor was measured to have a precise estimation of the volume. Each group contain 10 mouse models (*n* = 10 biological replicates in each group). (D_iii_) Kaplan–Meier survival curves of the groups exposed to PEC and NEC. There is no difference in the survival curves of non‐exposed (control) and NEC‐exposed groups (log‐rank test). These tests were performed in more than 400 mouse model which allocated in different groups randomly. (D_iv_) Average tumor volume reduction in the five groups of the study. All the data are shown as mean ± SD. Each group contain 10 mouse models (10 biological replicates for each group). (E) Comparative positron emission tomography of the exposed mouse models at the beginning of the PECT and 120 days after exposure. NEC, negative electrostatic charge; PECs, positive electrostatic charges

For determining whether external application of PECs (via a skin patch) inhibits malignant tumor growth in vivo, 1 × 10^6^/100 µl of 4T1 cancer cells (triple‐negative breast cancer mouse model cell line) were subcutaneously injected into the flank region of female BALB/c mouse models, followed by maintenance until the tumor volume reached ~800 mm^3^. Positively charged metallic (Al or Cu foils) flexible patches were externally attached on top of their skin at the tumor site using biological tapes (anti‐allergic surgical tape, Micropore Company) (Figure 3A). RECG does not generate any potentially lethal electrical currents^41^ (i.e., lower than 15 µA in 100 kV). Due to charge leakage between the mouse body and the ambient environment, the depleted current was tested before the exposure. This was done to be ensured that the electrostatic charges was continuously accumulated in desired region. In this way, the rate at which the tuned RECG accumulates suffiecent charges to compensate the depletion of charges from the patch and mouse body into the ambient environment. 1 and 5 kV electrostatic voltages were chosen as the low (16 nC) and high (90 nC) levels of PECs to be applied to the selected groups of mouse models, respectively. Exposure to PECs lasted approximately 12 days (tested by an electrostatic charge meter, MEECH, 983V2, Figure S2).

Images taken from the treated mouse models revealed a significant reduction in tumor size when the PECT voltage was increased to 5 kV (Figure 3B). Tumor degradation in PECs‐treated mouse models demonstrated a strong relationship between tumor degradation rate and charge intensity (Figure 3B). After approximately 10 days of continuous exposure to the RECG with power of 1 kV (16 nC), the mouse models treated with PECT^1^ became tumor‐free (Figure 3B). Sonography images also confirmed that the PECT‐induced tumor destruction is intensity‐dependent, with no tumor recurrence during the 120‐days follow‐up period (Figure 3C). It was surprising to see that the tumor size in mouse models exposed to PECT (5 kV cohort) decreased by approximately a quarter in <5 days (Figure 3C) without affecting other organs such as muscles, skin, brain, or kidneys (Figure S3). After approximately 2 weeks, even intermittent PECT (12 h per day, 5 kV) resulted in complete tumor destruction (Figure 3C). The results indicated that mouse models had been exposed to at least one course of PECT had completely disappeared tumors, whereas those who were withdrawn from the PECT and their treatment was not completed had non‐degraded tumors. Post‐exposure survival was approximately 1 year in completely treated mouse models but <60 days in the withdrawn cohort (Figure 3D_i_).

The tumor size reduction rate in PEC‐exposed mouse models was 160–170 mm^3^ per day at a voltage of 5 kV (Figure 3D_ii_). This degradation was observed only in the group exposed to PECs, whereas the group treated with NECs (at the same RECG power) had a tumor growth rate comparable to that of the non‐treated control group, with an expiration time of fewer than 35 days (Figure 3D_ii_,D_iii_). Furthermore, PET analysis revealed that after 120 days, tumors in the group exposed to continuous PECT at voltages of 1, 2, and 5 kV vanished entirely with no recurrence (Figure 3E). It is worth noting that PECT at high or low voltages had no adverse effect on the metabolism or structure of vital organs in mouse models (Figure S3).

Complementary tests, such as blood characterization, revealed no evidence of inflammation, ionic perturbation, or enzymatic dysfunction in the typical mouse models exposed to PECT compared to the control‐unexposed group. Specifically, vital ions such as Na, K, and Ca did not change significantly in the exposed mouse models, indicating that PECT is safe for the animals’ ionic balance (evaluated using one‐way ANOVA discussed in Figure S3).

### 3.3 Comparative histopathological analyses of PEC‐treated and control tumorized mouse models

**FIGURE 4:**
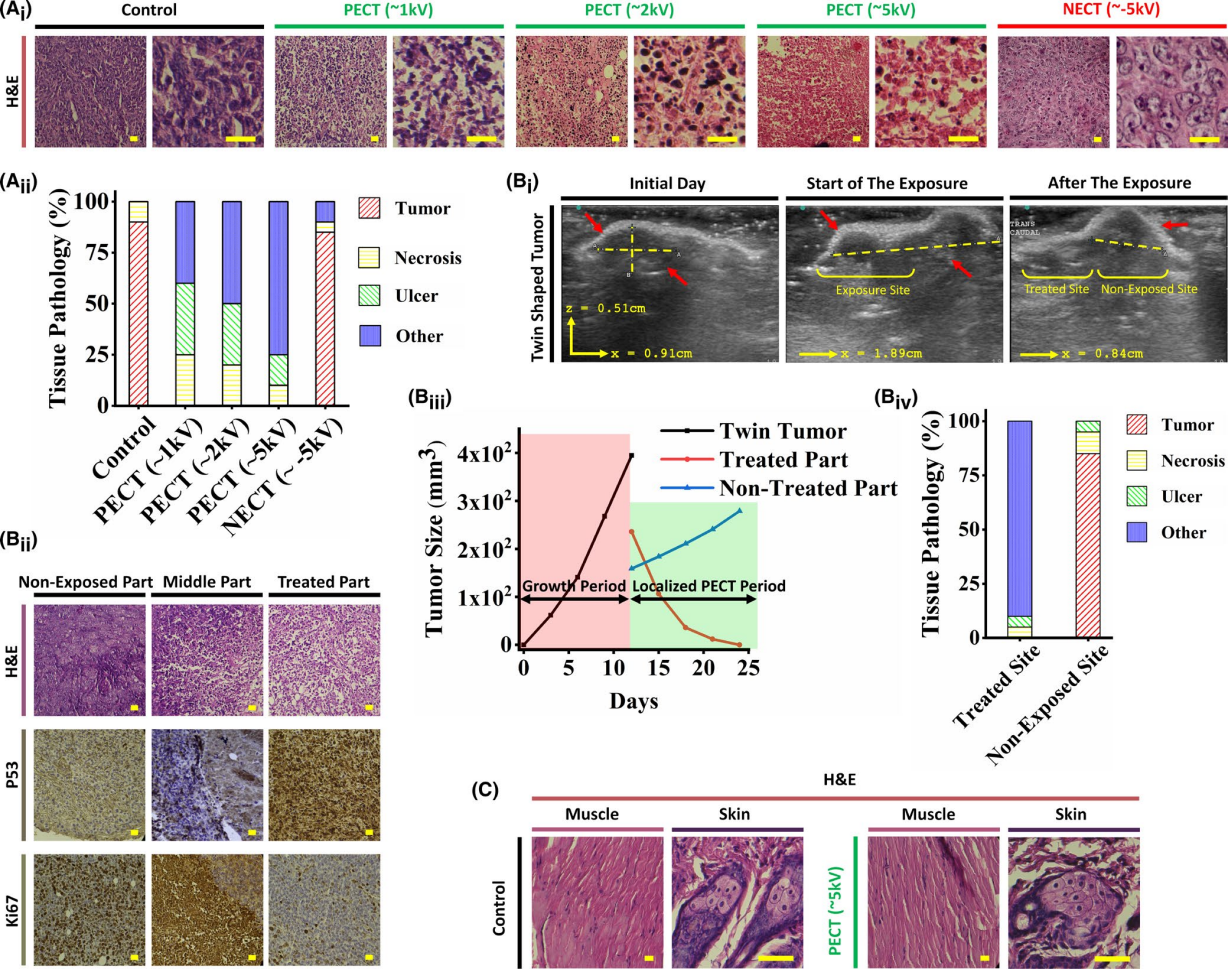
(A_i_) Histopathological analysis of the PEC‐exposed tumor sections. Pyknotic nuclei of the PECT samples show its effects. However, there is no observable change in the NECT sample in comparison to the PECT sample. As can be seen, the stroma of all exposed samples remains unaffected, demonstrating the non‐destructive effect of PECT on normal tissues. The scale bars are set to 5 µm. Each group contain 10 mouse models (10 biological replicates for each group). (A_ii_) Histopathological components of the analyzed PECT and NECT samples. The tumoral tissue has remained unchanged in both NECT and control samples. These include normal regional tissue, vascular channels, normal stromal/connective tissue, and fatty tissue. Each group contain 10 mouse models (10 biological replicates for each group). Figure S7A depicts tissue pathology components in detail. (B_i_) Ultrasonography images of a twin‐shaped tumor with PEC exposure to the left lump. Due to the identical structure, physiology, origin, and even location of such tumors, this twin‐shaped tumor provided an excellent model for studying the effects of PECT on exposed and non‐exposed tumor tissues. As can be seen, the non‐exposed part maintained tumor growth, whereas the exposed part suppressed tumor growth. (B_ii_) The H&E images of the half‐exposed twin tumor demonstrate the presence of apoptotic cells in the exposed region and unaffected cancer cells in the non‐exposed region. IHC images based on P53 and Ki67 indicate apoptosis and proliferation, respectively, only in exposed and non‐exposed regions. The scale bars are set to 5 µm. (B_iii_) Size alterations timeline in two periods of growth and PECT. Throughout the growth period, the total size of both lumps was calculated. For achieving the effects of PECT, each lump was sized separately following the PECT application. Significant size reduction and complete disappearance of the exposed lump compared to the non‐exposed hump clearly demonstrate PECT's localized effect on cancerous regions. (B_iv_) Pathological tissue components of the mentioned lumps. It is noteworthy that the components of the non‐exposed lump and the control group are identical. However, there is no evidence of cancerous tissue in the exposed lump. These include normal regional tissue, vascular channels, normal stromal/connective tissue, and fatty tissue. (C) Pathological images of control and exposed (~5 kV) mouse models depict no trace of apoptosis or necrosis in the regions exposed to PECT. The cells maintained their natural morphology and assemblies. The scale bars are set to 5 µm. PEC, positive electrostatic charge

Histopathological examinations of PEC‐treated mouse model tumors revealed a significant increase in apoptosis (Figure 4A_i_). Highly condensed nuclei, and dubbed pyknotic nuclei^42^ are visible in H&E images as the specification of exposed tumor cells. These are cells that have had their internal apoptotic pathways activated by PECT. Cytoplasmic degradation is more prevalent in malignant tissues treated with higher PEC intensities (90 nC, ~5 kV) (Figure 4A_i_,A_ii_). Since the exposed tumor tissue matrix remained unaffected, any possibility of necrosis was ruled out.^43^

PECT was applied to a single lump of a twin‐shaped tumor in a mouse model to ensure selective growth suppression and apoptosis in PEC‐exposed tumor cells (Figure 4B_i_). Only the exposed lump exhibited degradation (Figure 4B_i_). Additionally, pathological examinations in conjunction with IHC assays for P53 and Ki67 (as apoptotic^44^ and proliferative markers,^45^ respectively) confirmed selective tumor apoptosis in the exposed lump (Figure 4B_ii_,B_iv_). Furthermore, H&E examinations of the skin and muscles revealed no pathological effects in areas had been directly exposed to PECT (Figure 4C).

**FIGURE 5:**
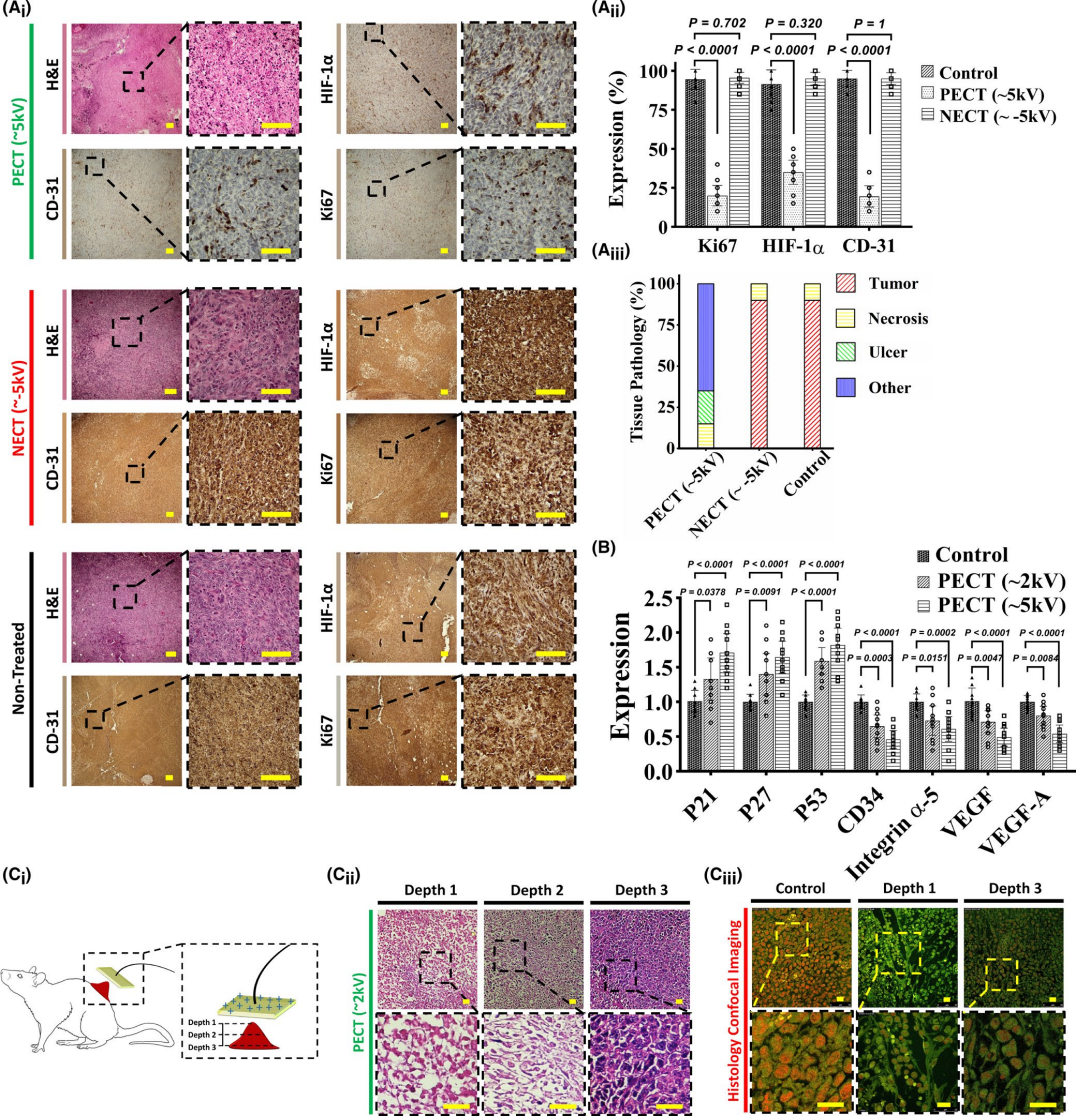
(A_i_) H&E and IHC analyses of three mouse model cohorts: control (non‐exposed), NECT, and PECT. Groups with exposure to PECT show pyknotic cancer cells with normal stroma in the H&E sections. HIF‐1α, CD31, and Ki67 IHC sections indicated the downregulation of hypoxia, angiogenesis, and proliferative markers, respectively. Cancerous cells have a high density and an irregular shape and structure in non‐exposed and NECT H&E sections, indicating that NECT does not affect cancerous tissues. Overexpression of HIF‐1α, CD31, and Ki67 IHC markers shows high levels of metabolism, angiogenesis, and proliferation in both non‐exposed and NECT samples. The scale bars are set to 20 µm. Each group contain 10 mouse models (10 biological replicates for each group). (A_ii_) Quantitative results of the expressions show the downregulation of HIF‐1α, CD31, and Ki67 in PECT samples compared to NECT and non‐exposed ones (independent *t*‐test). All the data are shown as mean ± SD. Each group contain 10 mouse models (10 biological replicates for each group). (A_iii_) Tissue components of the histological analysis of PECT, NECT, and non‐exposed samples. These include normal regional tissue, vascular channels, normal stromal/connective tissue, and fatty tissue. Figure S7B depicts tissue pathology components in detail. (B) RT‐PCR results for the three groups, that is, Control, PECT (~2 kV), and PECT (~5 kV). The overexpression of P21, P27, and P53 near the downregulation of CD33, integrin α5, VEGF, and VEGF‐A in exposed groups indicated that PECT induced detachment, followed by apoptosis, in the malignant tumor (independent *t*‐test). All the data are shown as mean ± SD. Each group contain 10 mouse models (10 biological replicates for each group). (C_i_, C_ii_) Schematic and comparative histological images between the surface and the deepest part of the tumor post‐exposure (~5 kV) with a primary depth of 1.5 cm indicated complete apoptosis and proliferation for these two regions, respectively. Depth‐dependent histopathological images of the exposed tumor indicated the progressed apoptotic region in the lower depth. It is worth noting that the tumor's deepest part measures 6 mm, up from 3 cm before initiating PECT. Hence, more than 2.4 cm of the tumor (in‐depth) was destructed. The scale bars are set to 5 µm. (C_iii_) Confocal microscopy of control and exposed (~5 kV) tumors. Immunofluorescent images of the non‐exposed and exposed surfaces and the depth (4 mm) of the exposed tumor revealed a non‐deformed shape, a severely deformed shape with condensed cytoplasm, a deformed shape with a decreased intensity, respectively. PEC exposure induced apoptosis to a depth of 4 mm in the tumor. The scale bars are set to 5 µm

IHC analyses showed the extreme downregulation of CD31, HIF‐1α, and Ki67, as glycolytic‐based angiogenesis,^46^ hypoxia‐based metabolic,^39^ and proliferative markers, in PEC‐treated tumors, respectively (Figure 5A_i_). In contrast, both control‐ and NEC‐exposed tumors exhibited no evidence of apoptosis/necrosis and also significantly expressed all three cancer‐supporting IHC markers (Figure 5A_i_). This may imply that the accumulation of PECs would disrupt hypoxia glycolysis, the primary metabolic pathway associated with cancer.

Quantitative real‐time PCR analysis revealed that the P21, P16, and P53 apoptosis‐related transcriptomes^47^ were significantly upregulated just in PEC‐exposed tumors and showed direct correlation with the intensity of PECs (Figure 5B). Additionally, adhesion and spreading‐associated transcriptomes^48^, ^49^ such as integrin α5, VEGF, VEGF‐A, and CD34 genes were downregulated only in PEC‐exposed tumors (Figure 5B).

It is worth noting that PECT's efficacy in annihilating tumors was depth‐dependent, owing to the inverse relationship between the strength of the electrostatic potential and the distance from the surface (Laplace's potential equation^50^) (Figure 5C_i_). H&E (Figure 5C_ii_) and confocal laser scanning images (Figure 5C_iii_) taken from various depths of the PEC‐treated mouse model tumor (32 nC, ~2 kV for 3 days) revealed decreased apoptotic induction in deeper regions of the tumor.

### 3.4 Comparison between the remission of PECT, RT, and ChT in mouse models

**FIGURE 6:**
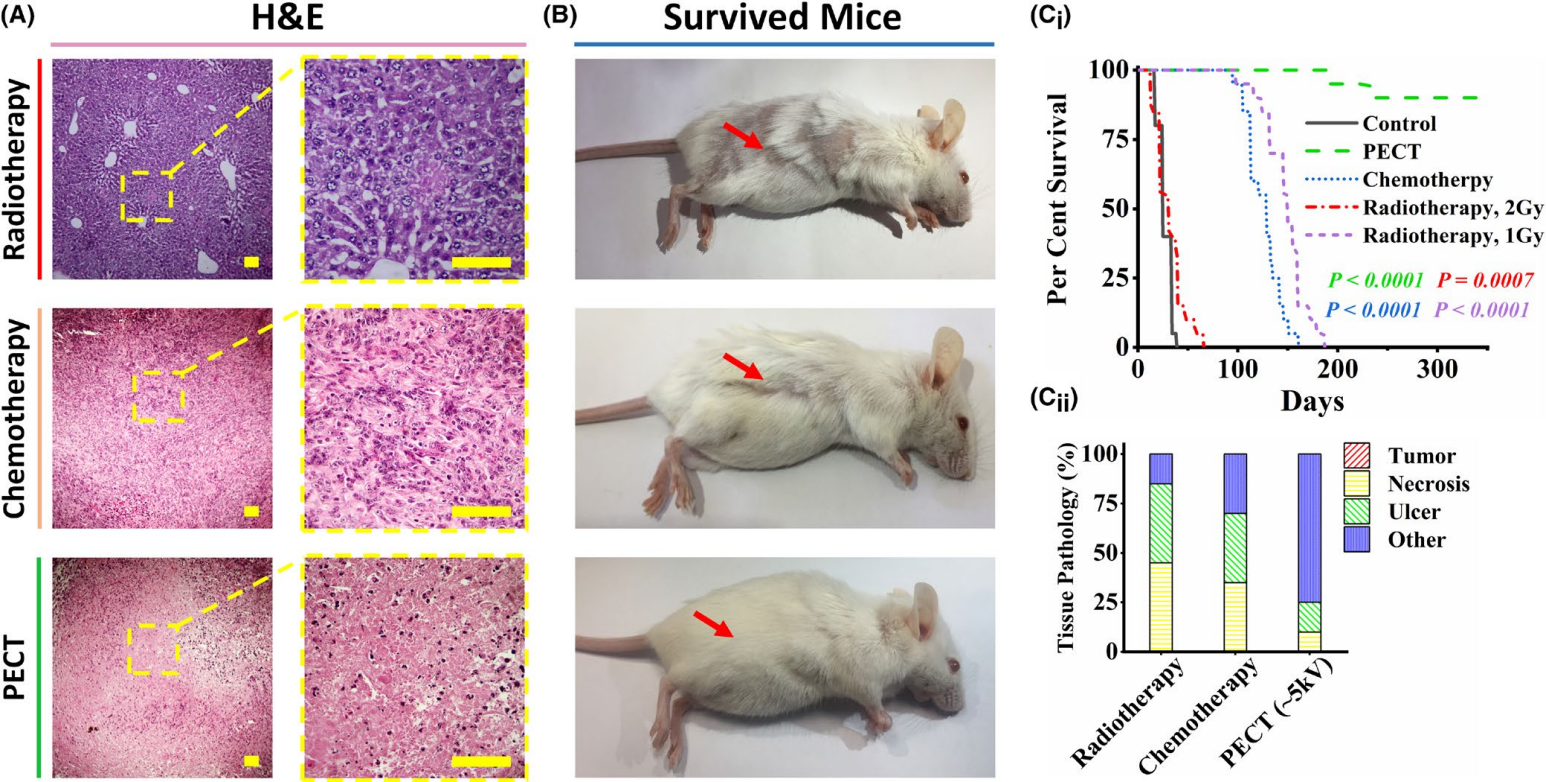
Comparative investigation of radiotherapy (RT), chemotherapy (ChT), and PECT effects on 4T1 tumorized mouse models. Five groups of mouse models were randomly chosen to study and compare the effects of two standard cancer treatment approaches (radiotherapy and chemotherapy) and PECT. Each group contain 10 mouse models (10 biological replicates for each group). These were designated as the control, PECT, chemotherapy, and radiotherapy groups (in two groups of 1 and 2 Gy, two standard radiotherapy regimens for mouse models). (A) Histopathological images of treatments indicated severe necrosis profiles in tumors of all treated cohorts. The scale bars are set to 20 µm. (B) Four weeks after complete remission, the optical images of the survived mouse models in the mentioned approaches showed severe hair loss, weight loss, and local burning of the RT groups. Hair loss and weight loss can also be observed in the ChT group. However, there is no sign of hair loss or weight loss after PECT (even in poor mobility conditions due to the patches’ location). (C_i_) Kaplan–Meier survival curves of the mentioned groups. The survival rate of the RT group (2 Gy) is almost similar to that of the control group. The RT group (1 Gy) and the ChT group showed poor survival rates (log‐rank test). (C_ii_) Tissue pathology components of the tumor location of the treated mouse models in each group. Figure S7C depicts tissue pathology components in detail

In mouse models with 4T1 tumors, the efficacy and side effects of PECT were compared to those of RT and ChT. PECT demonstrated superior therapeutic efficacy in comparable mouse models with significantly fewer adverse effects (Figure S3). Five groups of mouse models (each containing 10 mouse models) were randomly chosen to study and compare the effects of two standard cancer treatment approaches (RT and ChT) and PECT. These were designated as the control, PECT, ChT, and RT groups (in two groups of 1 and 2 Gy as two standard RT regimens for mouse models). Histopathological images of post‐treatment tumor residues revealed severe necrosis in RT and PEC‐treated sections, as well as in ChT mouse models treated with PTX (dose and procedures are described in the method section) (Figure 6A). Four weeks after complete remission, optical images of the survivors in the approaches mentioned above revealed significant hair loss, weight loss, and localized burning in the RT groups (Figure 6B). In the ChT group, moderate hair loss and weight loss were also observed. In comparison, no evidence of hair loss or weight loss was observed following PECT. The Kaplan–Meier survival curves for the groups mentioned above indicated that PEC‐treated mouse models had significantly higher survival rates than ChT and RT cohorts (Figure 6C_i_). While over 80% of the PEC‐treated mouse models remained alive after 300 days, the RT (1 Gy) and ChT groups had significantly lower survival rates (log‐rank test, *p* < 0.0001), with none remaining alive after 200 days. Additionally, the survival rate of the RT group (2 Gy) was remarkably lower than other mentioned groups (Figure 6C_ii_). Comparing the tissue pathology components of the mouse models in each group revealed that the PEC‐treated animals had the least amount of ulcer and necrosis, demonstrating their efficacy and higher recovery rate (Figure 6C_ii_). Figure S7C depicts tissue pathology components in detail.

While PECT may be promising as a complementary treatment for solid malignant tumors, some limitations must be addressed before conducting human trials. While it significantly affects superficial tumors, most human solid metastatic tumors are located in deeper tissues such as the lung, pancreas, and liver bed. As a result, the possibility of increasing the PEC voltage or utilizing charge transfer assisted by wire guides must be considered. Additionally, ambient humidity may contribute to increased current leakage from the treating patch. Thus, it is critical to adjust the humidity of the treating room during PECT to avoid current leakage and provide an appropriate level of humidity for the patient's breathing. After obtaining the necessary certifications, our group will investigate all of these concerns to optimize the application of PECT in a human model study.

## 4 CONCLUSION

In summary, we discovered an intrinsic ability of pure PECs to induce selective apoptosis in cancer cells and used it to destroy malignant tumors in animal models with clinically validated results. PEC was used to treat human breast cancer cell lines and tumorized female mouse models. Histopathological examinations, cellular/molecular analyses such as flow cytometry, immunohistochemistry, real‐time PCR, and confocal imaging results revealed activation of apoptosis as well as suppression of proliferative markers in treated malignant cell lines and mouse model tumors.

This study presented impressive therapeutic effects on malignant tumors with no adverse effects on normal tissues, indicating that PECT may be a new therapeutic approach for metastatic malignant tumors combined with other treatments. It may shed new light on the bio‐electrical factors underlying cancer metabolism, allowing for the development of safe cancer treatment protocols. Comparing PECT's efficacy to that of radio/ChT in mouse models demonstrated the advantages of the procedure. Finally, to assess the efficacy of PECT in the human model, patients with various types of progressive metastatic solid tumors will be exposed to optimize the PECT protocols, and they will be followed for at least 6 months after obtaining the necessary certificates.

## CONFLICT OF INTEREST

The authors declare no potential conflict of interest related to this study. A US patent has been granted on the basis of this work by M.A., A.Z., and S.R.T. (Patent No.: US 10,806,945 B2).

## ETHICAL COMMITTEE CERTIFICATION

The University of Tehran animal ethics committee approved all the procedures. All the tests were conducted with the ethical code of IR.TUMS.VCR.REC.1397.354.

## Supporting information

Figures S1‐S7

Tables S1‐S3

Video S1

Video S2

## Data Availability

The data that support the findings of this study are available from the corresponding author upon request.

## ENDNOTE

^1^
In our definition when PECT was used in any in vivo either mouse models or human tests or trials in this article, it means one course of PECT which is equal to one period of sequential treatments up to referring the animal/patient for clinical follow‐up. Hence, each course of PECT maybe vary between 5 and 24 days with the voltage range between 0.1 and 50 kV depend on the nature of the case (mouse models or human) and the stage of it.
