# Supplementary material for: Positive electrostatic therapy of metastatic tumors: selective induction of apoptosis in cancer cells by pure charges
Source: Cancer Med. 2021 Oct 9;10(21):7475–91. doi: 10.1002/cam4.4267 (PMC8559484; doi:10.1002/cam4.4267)
Supplement: Supplementary file 1 — Figures S1‐S7 [file CAM4-10-7475-s002.docx]

Supplementary Information

**Positive Electrostatic Therapy of Metastatic Tumors; Selective Induction of Apoptosis in Cancer Cells by Pure Charges**


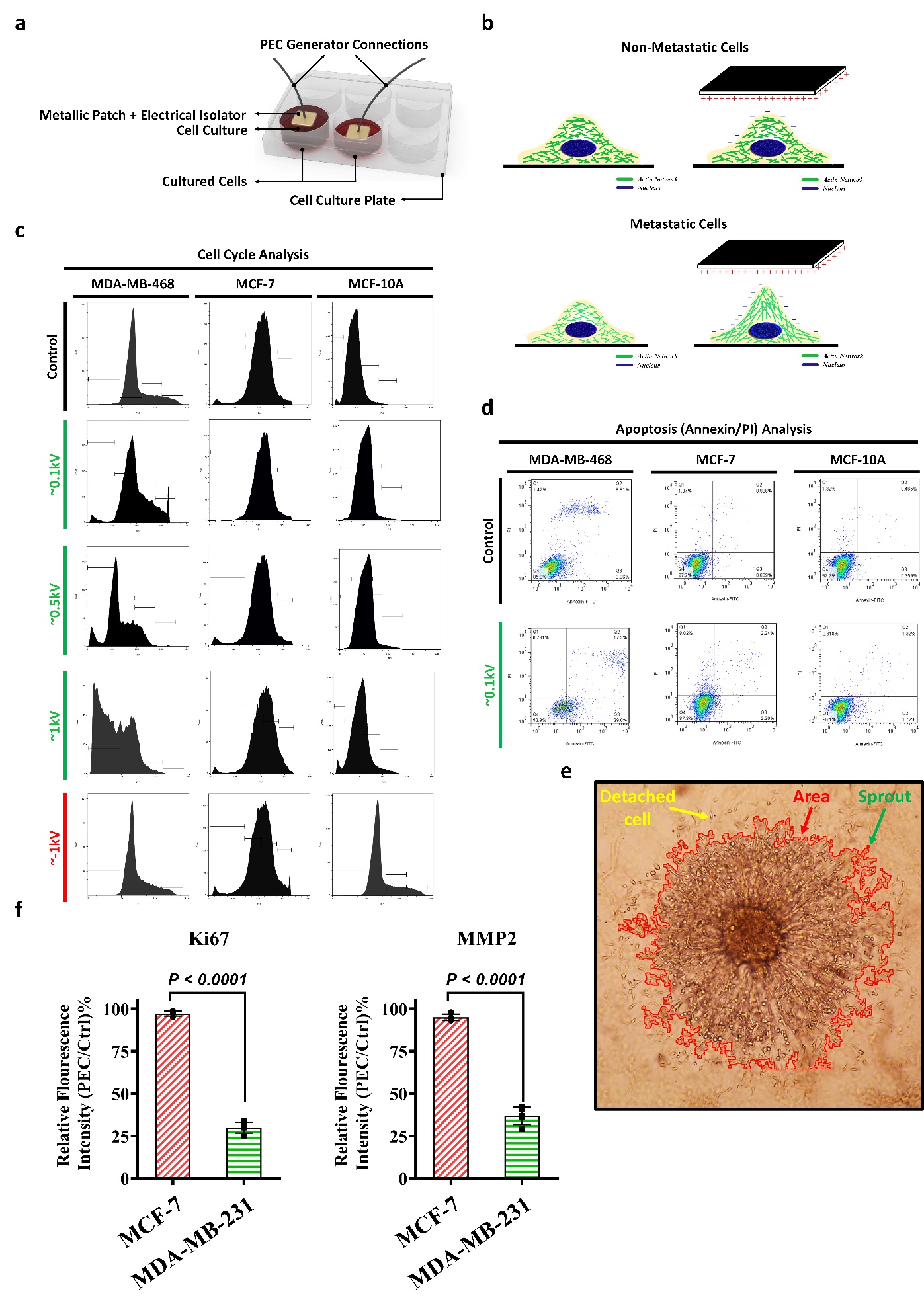


***Figure S-1,*** *(a) Schematic of the PEC applied on the cell lines. The metallic patch, connected to the PEC generator, was isolated via a thin layer of biocompatible electrical isolator (Polydimethylsiloxane, PDMS) to prevent the leakage of electrostatic charges and increase their accumulation. The isolated patches touch (tangent) the surface of the cell culture media. All the tests were conducted in the highest level of the cell medium (~2cm) in 6-well cell culture plates (b) Schematic of the tendency of the malignant cells to the source of positive charges, which resulted in their detachment from the substrate or ECM. (c) Cell cycle analysis of differently stimulated samples for the MCF-7, MCF-10A, MDA-MB-468 cell lines, representing Figure 1-a. (d) Annexin/Pi analysis conducted for control and PEC-exposed samples, representing Figure 1-d. The other exposed cell groups have not experienced a meaningful shift toward the right, as observed in the exposed metastatic cells. This shift indicates an increase in early apoptotic rates, implying the achievement of a significant selective destructive effect on metastatic cells. (e) A sample of image-processed spheroids. Area, sprouts, and detached cells are indicated in the figure. (f) Comparative normalized (per control) graphs of relative fluorescent intensity in Ki67 (proliferation) and MMP2 (invasion) markers depict a significant decrease in the proliferation and invasion expression of the exposed highly malignant cells (MDA-MB-231) (P < 0.0001, independent t-test). Each bar contains 5 biological replications of fluorescent staining.* *The data are shown as mean ± SD.*


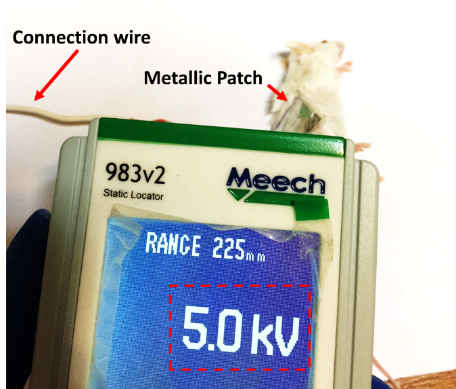


***Figure S-2,*** *Measuring the electrostatic potential applied on the tumorized mouse models using an electrostatic charge meter, MEECH, 983V2. An electrical isolated metallic patch was placed on the tumor to accumulate the electrostatic charge above the tumor. The isolated patch was connected to an electrostatic charge generator via a high voltage DC cable (Tokyo Shibaura Electric Co., Kawasaki) to decrease current leakage.*


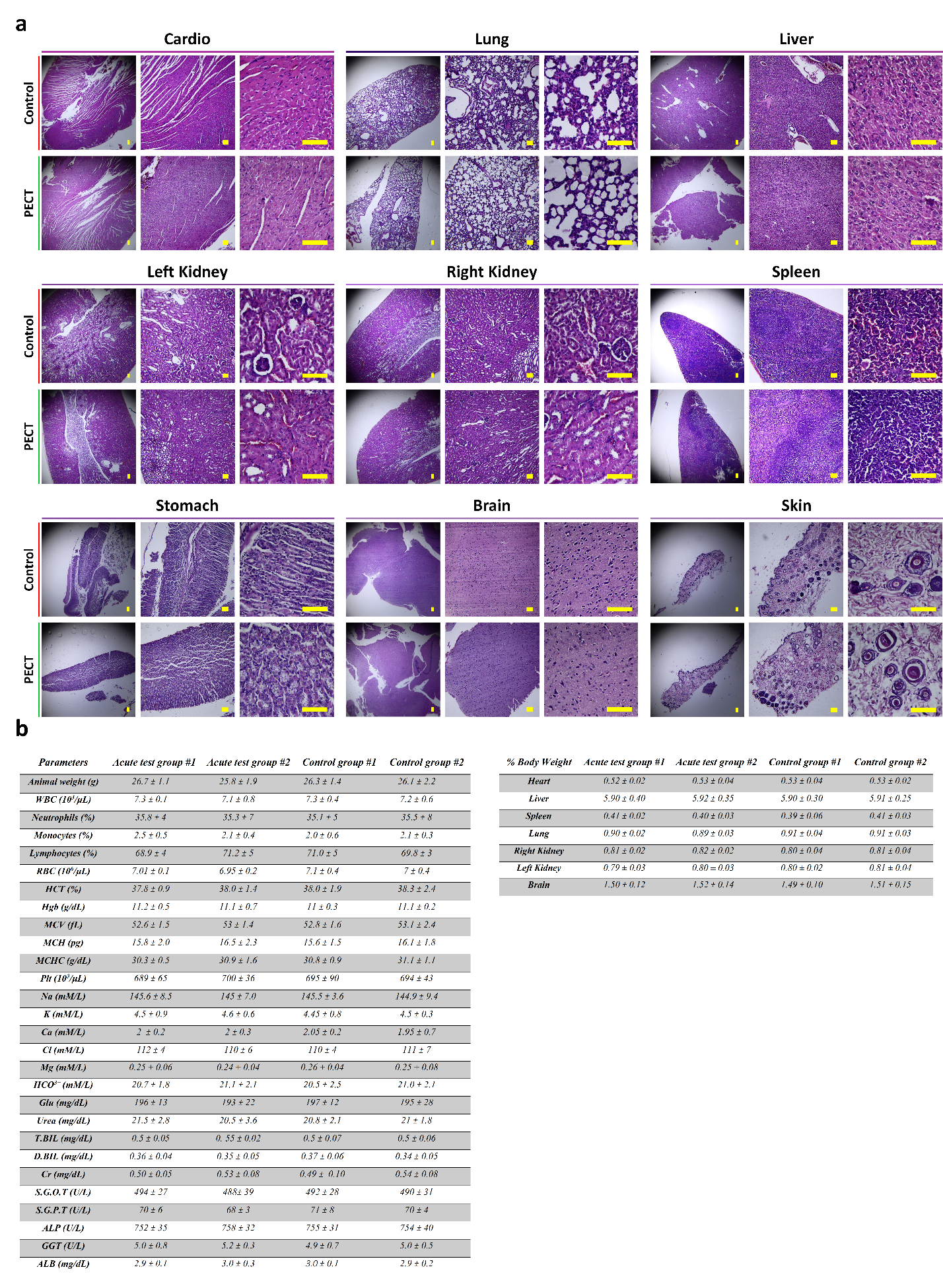


***Figure S-3,*** *In order to systematically investigate the destructive effects of PEC exposure, four groups of mouse models were randomly chosen for two cohorts of PEC exposure (case) and control. There were 10 mouse models in each group (10 biological replicates for each group). The exposed groups received PEC continuously at ~10 kV for 30 days. (a) Histopathological assays of PEC-exposed and control mouse models organs. Comparative analyses depict no histopathological effects on mouse models organs, even in acute conditions of PEC exposure. The scale bars are set to 20µm. (b) Blood characterization of PEC-exposed and control groups. They have been tested for 27 blood and 8 anthropometric factors. The values in each group have been tested with a one-sample Kolmogorov-Smirnov test, showing that the distribution of the values in each group is normal. The mean of the four groups was compared by one-way ANOVA in terms of each factor separately. The null hypothesis states no statistically significant difference between the four groups and is therefore not rejected. In other words, the difference in mean values between the case and control groups is not statistically significant (P> 0.05), which shows that there is no significant difference in serum and anthropometrics factors of the mouse models in the control and PEC group.*

**Van de Graff generator** is an electrostatic charge generator. Van de Graff includes some moving parts, and their movement results in the accumulation of charges in a hollow metal sphere on top of the device. A rubber belt and two rollers with different materials (the upper one is metal) are the moving parts. The interaction and contact between the rollers and the rubber belt result in electron transfer from one material to another. This electron transfer occurs due to the triboelectric effect, which explains how materials exchange electrons following a brief contact. A sharp metal blade transfers an electron from the belt to the metal sphere.

The contact between the rubber belt and the bottom roller (polytetrafluoroethylene, PTFE) makes the belt's inner side negative and the belt positive. The belt transfers the positive charge to the upper roller, and by accumulating the positive charges in the upper metal roller, charges will be induced in the sharp blade, thus accumulating in the metal sphere.


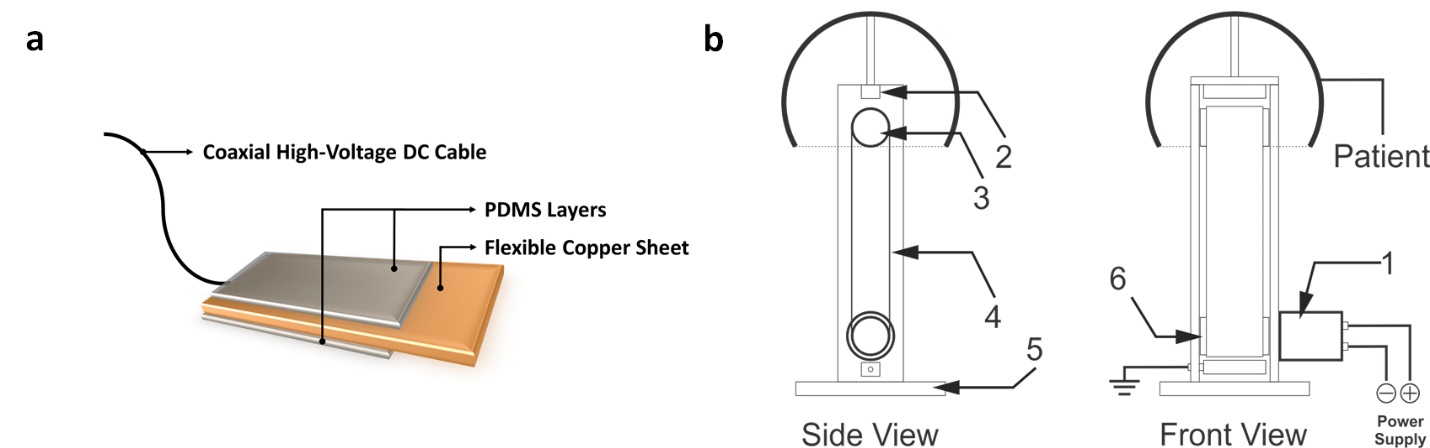


***Figure S-4,*** ***(a)*** *Schematic of the flexible patch used to apply the electrostatic stimulation.* ***(b)*** *Side and front view schematics of a Van de Graff generator: 1. DC electric motor, 2. Upper sharp blade, 3. Upper metal roller, 4. Rubber belt, 5. Grounded stage, and 6. Lower PTFE roller. The metal sphere is labeled as the electrostatic charge. Additionally, the lower sharp blade is grounded to prevent the accumulation of negative charges, reducing the effectiveness of charge accumulation on the metal sphere.*

The electric potential of a point charge is:

$$V=\frac{\mathrm{kq}}{r}\to q=\frac{\mathrm{rV}}{k},$$

where q is the point charge, r is the distance from the charge, and k is a constant equal to

$$k=8.99\times{10}^{9} \frac{N.m^{2}}{C^{2}}.$$

Therefore, if we have the electric potential (using an electrostatic charge meter, MEECH, 983V2) and the distance from the charge (as we assume the center of the sphere on the top of the Van de Graff generator to be the location of the total charge and the radius of the sphere to be the distance to solve the equation without any complexity), the total charge will be:

$$q= \frac{\mathrm{rV}}{8.99 \times{10}^{9}}.$$

Hence, if ΔT is the tumor size (mm^3^) destruction per day by a distinct applied charge, then:

$$\Delta T=\frac{t_{n}-t_{0}}{n}.$$

Therefore, the tumor destruction rate in 24 hours per the applied charge (nanocoulomb) will be:

$$D=\frac{\Delta T}{q}$$

Figure S5 depicts the tumor destruction rate per nanocoulomb in the treated mouse models.


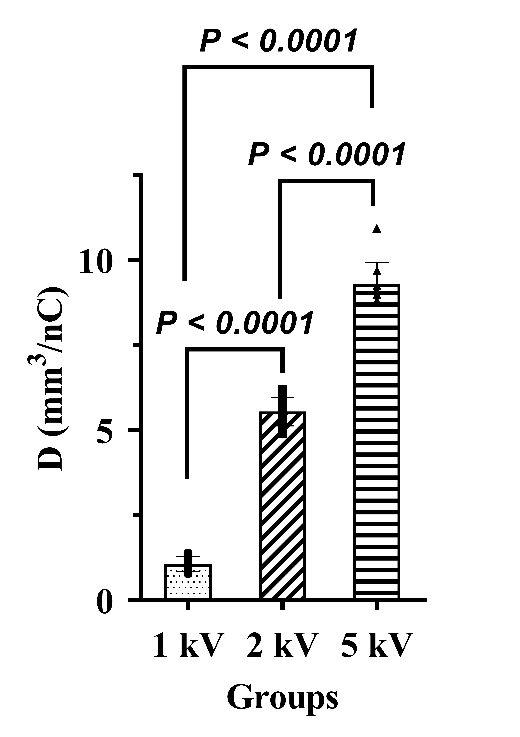


***Figure S-5****, Tumor destruction rate per applied charge (nanocoulomb) during a day of PECT (P < 0.0001, one-way ANOVA, followed by Tukey HSD). The data are shown as mean ± SD.* *Each group contain 10 mouse models (10 biological replicates).*


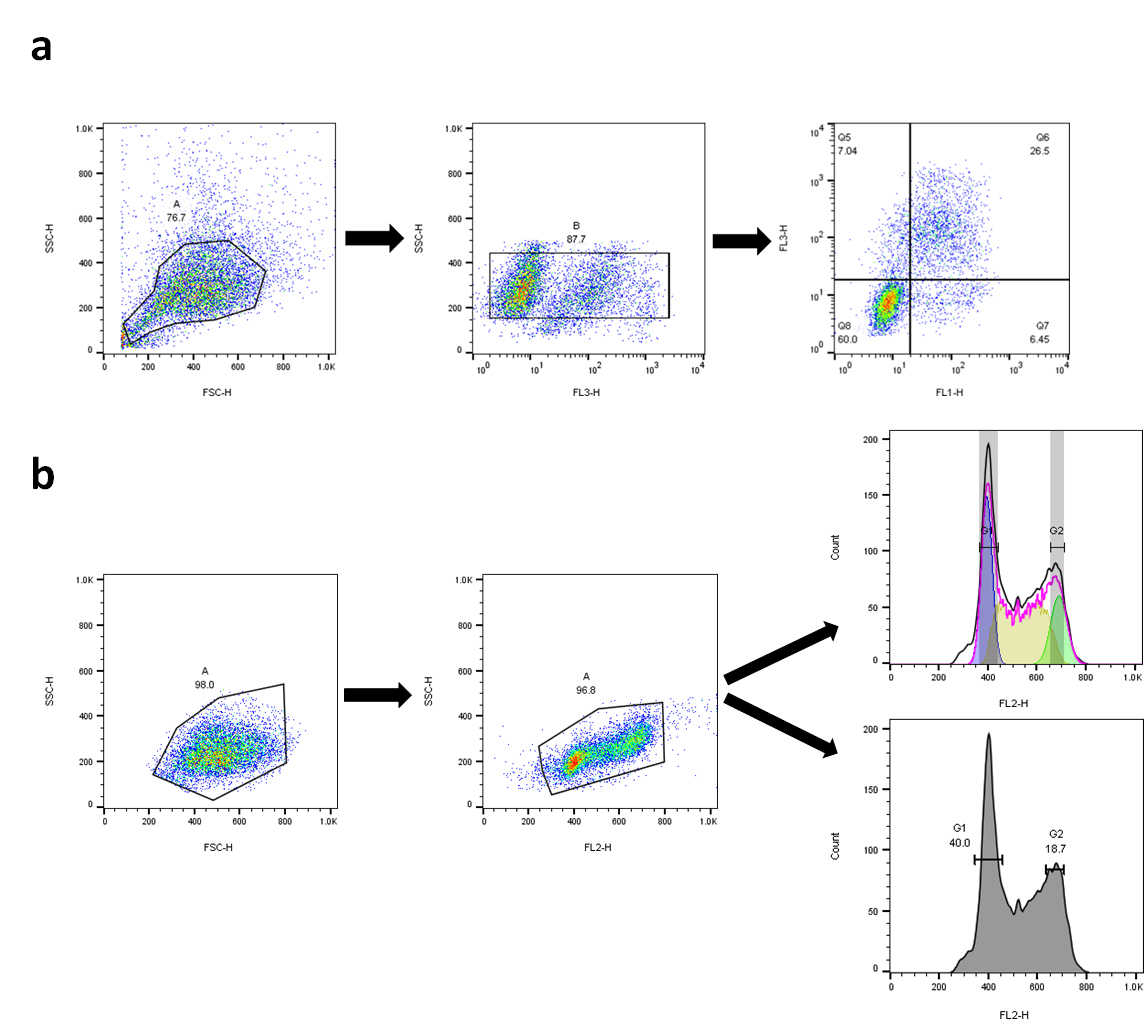


***Figure S-6,*** ***(a)*** *Apoptosis flow cytometry analysis. An instance of gating strategy used to indicate cells in apoptosis, staining with Annexin-V and PI.* ***(b)*** *Cell-cycle flow cytometry analysis. An instance of gating strategy used to imply cell cycle.*


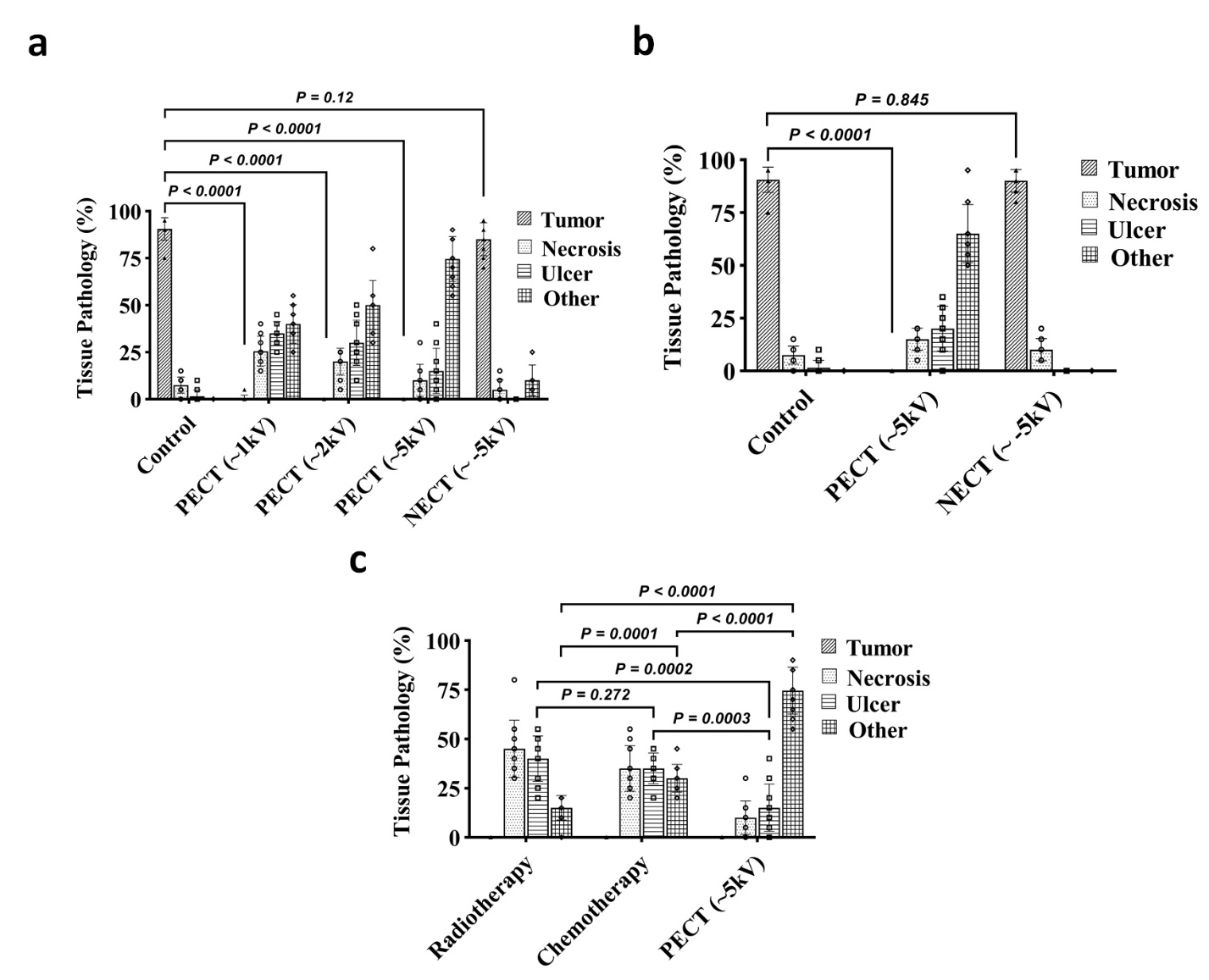


***Figure S-7****, Tissue pathology components. Each group contains 10 mouse models (10 biological replicates for each group). P-values obtained by independent t-test. The data are shown as mean ± SD.* ***(a)*** *Tissue pathology components of figure 4-a_ii_.* ***(b)*** *Tissue pathology components of figure 5-a_iii_.* ***(c)*** *Tissue pathology components of figure 6-c_ii_.*
