# Supplementary material for: Positive electrostatic therapy of metastatic tumors: selective induction of apoptosis in cancer cells by pure charges
Source: Cancer Med. 2021 Oct 9;10(21):7475–91. doi: 10.1002/cam4.4267 (PMC8559484; doi:10.1002/cam4.4267)
Supplement: Supplementary file 2 — Tables S1‐S3 [file CAM4-10-7475-s001.docx]

| Gene | Primer Sequence |
| --- | --- |
| P21 | F (CCAGCCTGACAGATTTCTAT)  R (GC CCTACCGTCCTACT) |
| P27 | F (AGGAGAGCCAGGATGTCAGC)  R (CAGAGTTTGCCTGAGACCCAA) |
| P53 | F (CTACAAGAAGTCACAGCACAT)  R (CCATCACCATCGGAGCAG) |
| CD34 | F (GCTTACACATCATCTTCTGCT)  R (CCAACCTCACTTC TCGGA) |
| Integrinα5 | F (TCACCTATGGCTATGTCAC)  R (AATCGGCTGAACTCATCTT) |
| VEGF | F (GACAGAAGGAGAGCAGAAGT)  R (AATCGGAC GGCAGTAGC) |
| VEGFRa | F (CCTGTCCAACTACCTCAA)  R (CTGTA ATCCTCGTCTCCTT) |
| GAPDH | F (AGCCTCAAGATCATCAGCAATG)  R (ATGGACTGTGGTCATGAGTCCTT) |

***Supplementary Table 1****, the primers used for SYBR Green real-time RT-PCR.*

**Mouse models endpoints:**

All the animal procedures and endpoints were performed in accordance with the guidelines for Animals in Research and Reporting In Vivo Experiments (ARRIVE) of Tehran University of Medical Sciences.

Different endpoints were determined including death or moribundity, complete remission, and partial remission. Moribund conditions described as clinically irreversible conditions leading inevitably to death.

The control groups set for each study separately.

***Supplementary Table 2****, Implemented endpoints in the mouse model groups with criteria, signs, and symptoms.*

| Endpoints | Criteria, Signs, and Symptoms | Applied Groups |
| --- | --- | --- |
| Death or moribundity | 1. Lack of response to stimulation. 2. Immobility. 3. Inability to eat or drink. 4. Abnormal posture. 5. Rough hair coat. 6. Head tucked into abdomen. 7. Exudates around eyes and/ or nose. 8. Skin Lesions. 9. Abnormal breathing. 10. Self- mutilation. 11. Extensive ulcerative tumors, necrotic, and infected (in non-treated groups). 12. All the clinical signs and observations that have the potential to cause pain and distress in animal models (described in Supplementary Table 3). | All the groups of Figure 3, treated (PECT and NECT) and non-treated (control).  All the groups of Figure 6 (radiotherapy, chemotherapy, PECT). |
| Complete remission in treated groups | 1. Radiological symptoms (Sonography, PET Scan). 2. Extensive ulcerative tumors, necrotic, and infected (in non-treated groups). 3. All the clinical signs and observations that have the potential to cause pain and distress in animal models (described in Supplementary Table 3). | All the groups of Figure 4-a, treated (PECT and NECT) and non-treated (control).  Figure 4-b, left lump as treated (PECT) and right lump as non-treated (control).  All the groups of Figure 5-a&b, treated (PECT and NECT) and non-treated (control).  All the groups of Figure 6 (radiotherapy, chemotherapy, PECT). |
| Partial remission | 1. Three days of PECT exposure. 2. Decreased tumor size. 3. All the clinical signs and observations that have the potential to cause pain and distress in animal models (described in Supplementary Table 3). | Treated group of Figure 5-c. |

***Supplementary Table 3****, Clinical observations (Montgomery, C.A. Jr. (1990), Cancer Bulletin 42:230-237).*

| Clinical Parameter | Observations |
| --- | --- |
| General appearance | Dehydration, decreased body weight, missing anatomy, abnormal posture, hypothermia, fractured appendage, swelling, tissue masses, prolapse, paraphimosis. |
| Skin and fur | Discoloration, urine stain, pallor, redness, cyanosis, icterus, wound, sore, abscess, ulcer, alopecia, ruffled fur. |
| Eyes | Exophthalmos, microphthalmia, ptosis, reddened eye, lacrimation, discharge, opacity. |
| Nose, mouth, and head | Head tilted, nasal discharge, malocclusion, salivation. |
| Respiration | Sneezing, dyspnea, tachypnea, rales. |
| Urine | Discoloration, blood in urine, polyuria, anuria. |
| Feces | Discoloration, blood in the feces, softness/diarrhea. |
| Locomotor | Hyperactivity, coma, ataxia, circling, muscle, tremors. |
